# Isavuconazole: Thermodynamic Evaluation of Processes Sublimation, Dissolution and Partition in Pharmaceutically Relevant Media

**DOI:** 10.3390/molecules26164759

**Published:** 2021-08-06

**Authors:** Marina Ol’khovich, Angelica Sharapova, Svetlana Blokhina, German Perlovich

**Affiliations:** G.A. Krestov Institute of Solution Chemistry, Russian Academy of Sciences, 1 Akademicheskaya Street, 153045 Ivanovo, Russia; omv@isc-ras.ru (M.O.); savs@isc-ras.ru (A.S.); svb@isc-ras.ru (S.B.)

**Keywords:** isavuconazole, sublimation, solubility, protolytic properties, Hansen solubility parameter, partition, thermodynamic functions

## Abstract

A temperature dependence of saturated vapor pressure of isavuconazole (IVZ), an antimycotic drug, was found by using the method of inert gas-carrier transfer and the thermodynamic functions of sublimation were calculated at a temperature of 298.15 K. The value of the compound standard molar enthalpy of sublimation was found to be 138.1 ± 0.5 kJ·mol^−1^. The IVZ thermophysical properties—melting point and enthalpy—equaled 302.7 K and 29.9 kJ mol^−1^, respectively. The isothermal saturation method was used to determine the drug solubility in seven pharmaceutically relevant solvents within the temperature range from 293.15 to 313.15 K. The IVZ solubility in the studied solvents increased in the following order: buffer pH 7.4, buffer pH 2.0, buffer pH 1.2, hexane, 1-octanol, 1-propanol, ethanol. Depending on the solvent chemical nature, the compound solubility varied from 6.7 × 10^−6^ to 0.3 mol·L^−1^. The Hansen s approach was used for evaluating and analyzing the solubility data of drug. The results show that this model well-described intermolecular interactions in the solutions studied. It was established that in comparison with the van’t Hoff model, the modified Apelblat one ensured the best correlation with the experimental solubility data of the studied drug. The activity coefficients at infinite dilution and dissolution excess thermodynamic functions of IVZ were calculated in each of the solvents. Temperature dependences of the compound partition coefficients were obtained in a binary 1-octanol/buffer pH 7.4 system and the transfer thermodynamic functions were calculated. The drug distribution from the aqueous solution to the organic medium was found to be spontaneous and entropy-driven.

## 1. Introduction

**Figure 1:**
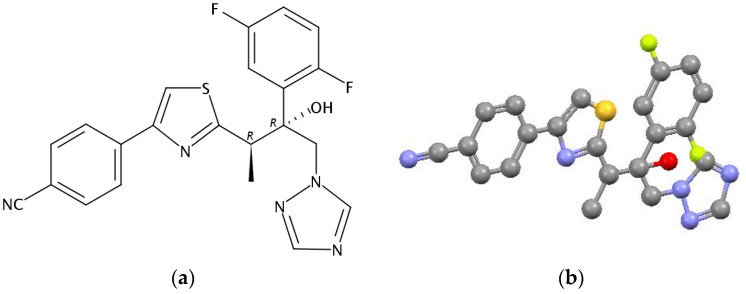
Chemical structure (**a**) and three-dimensional representation (**b**) of IVZ.

Invasive fungal infections, such as aspergillosis and the rather rare mucormycosis, remain the main cause of disease and death in immunocompromised patients [1,2,3,4,5]. The triazole class of antifungal drugs includes first-line preparations for treatment of diseases caused by a number of medicinally important strains of opportunistic fungi [6,7,8,9]. Isavuconazole (IVZ) is a new second-generation hybrid of thiazole and triazole with a wide spectrum of antifungal activity and favorable pharmacokinetic and pharmacodynamic profiles and is the only new antimycotic agent for systemic use that has been registered in the last 10 years. The isavuconazole structural formula is shown in Figure 1.

Isavuconazole is available for oral and intravenous administrations. There are now ongoing clinical trials (phase III) to determine isavuconazole effectiveness in the treatment of invasive candidiasis and aspergillosis. There are practically no data about the physicochemical characteristics of this new promising antimycotic drug. That is why the aim of our work is to study the fundamental pharmaceutically relevant properties of isavuconazole in solid state and solutions based on the data on sublimation, solubility and distribution of this compound in model biological media.

Solubility in pharmaceutically relevant media and ability to penetrate lipophilic membranes are the main parameters determining the availability of a drug compound and its pharmacological activity. Experimental data on solubility make it possible to identify effective therapeutic dosages and reduce side effects [10]. A temperature dependence of solubility can be used to carry out thermodynamic analysis that will make it possible to identify the intermolecular mechanisms of the dissolution process [11]. Additionally, the main thermophysical properties of drugs—melting temperature and enthalpy of solid–liquid transition—can be used to correctly determine the ideal solubility of a drug in various solvents [12].

When studying the solubility of compounds, it should be taken into account that this parameter depends on the solid state and energy of intermolecular solute–solvent interactions [13]. There are limited data about the thermodynamic functions of sublimation of the azole class of compounds in the literature [14]. Since there is no information about drug sublimation enthalpies, it is impossible to study solvation processes of pharmacophore molecules and evaluate the enthalpy of formation of these compounds by quantum-chemical methods [15].

Lipophilicity is responsible for pharmacokinetic behavior of drugs in the human body: penetration through the blood–brain barrier and gastro-intestinal tract walls and binding with enzymes. Lipophilicity is greatly affected by intermolecular interactions that, in turn, depend on structural factors [16]. Partition coefficients in the 1-octanol/water system are a common measure of lipophilicity of compounds and a necessary characteristic of drugs. Additionally, data on partition coefficients are useful for prediction of biological activity of compounds by computer simulation methods.

## 2. Results and Discussion

### 2.1. Thermophysical Properties

**Figure 2:**
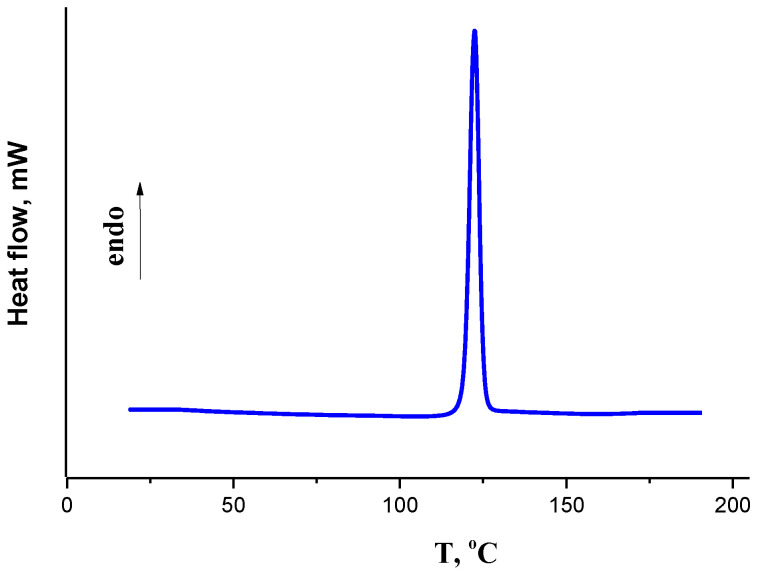
DSC curve of isavuconazole obtained by heating with rate 2 K·min^−1^.

The isavuconazole thermal stability was studied by the DSC method within the temperature range from 25 to 250 °C. The DSC curve shown in Figure 2 has an endothermic peak of melting at T_m_ (onset) = 392.75 ± 0.2 K. The drug melting enthalpy (∆_m_H), which equals 29.9 ± 0.5·kJ·mol^−1^, was calculated based on the area under the melting peak curve limited by the baseline. Uncertainties for melting parameters correspond to expanded uncertainty of the mean (0.95 confidence level). No additional phase transitions were found between the temperature of 298 K and the melting point of the studied compound, which confirms that there were no polymorphic modifications or hydrated forms.

### 2.2. Sublimation

#### 2.2.1. Experimental Results

The IVZ saturated vapor pressure within the temperature range of 365.15–383.15 K was measured by the method of gas carrier transfer and is shown in Table 1.

**Figure 3:**
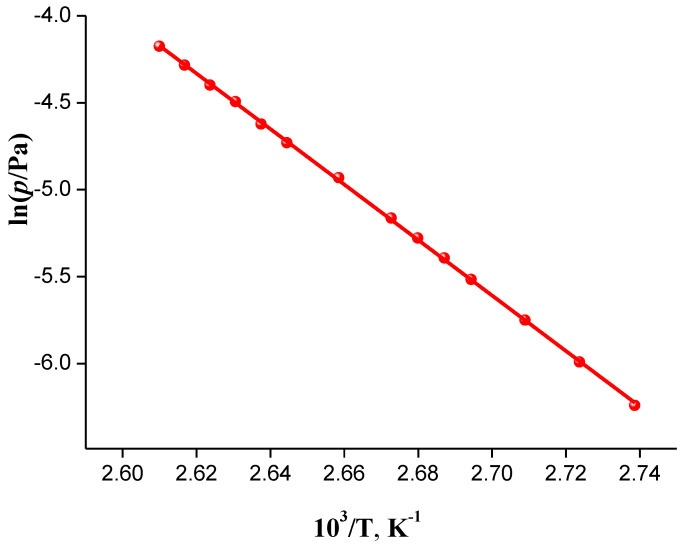
Plot of vapor pressure against reciprocal temperature of the compound studied.

Figure 3 presents the dependence of the saturated vapor pressure of the studied compound on reciprocal temperature that is described by the equation ln(*p*/Pa) = (37.48 ± 0.17) − (15,959 ± 66)/T with the correlation coefficient R = 0.9999. For the calculation, we used the assumption that isavuconazole molecules in the gas phase were in the monomolecular state, which was confirmed by the low saturated vapor pressure values (0.001–0.01 Pa) within the respective temperature range and linearity of the vapor pressure temperature dependence.

The thermodynamic functions of sublimation were calculated as the fitting coefficients of the Clarke and Glew equation [17]:

(1) $$R\ln\frac{p}{p^{0}}=\frac{\Delta_{cr}^{g}G_{m}^{{}^{\circ}}(\theta )}{\theta }+\Delta_{cr}^{g}H_{m}^{{}^{\circ}}(\theta )(\frac{1}{\theta }-\frac{1}{T})+\Delta_{cr}^{g}C_{p,m}^{{}^{\circ}}(\theta )(\frac{\theta }{T}-1+\ln\frac{T}{\theta })$$

where $\Delta_{cr}^{g}H_{m}^{{}^{\circ}}(\theta )$ is the standard molar sublimation enthalpy, $\Delta_{cr}^{g}G_{m}^{{}^{\circ}}(\theta )$ is the standard molar Gibbs energy of sublimation, and $\Delta_{cr}^{g}C_{p,m}^{{}^{\circ}}(\theta )$ is the difference between the constant pressure standard molar heat capacity $C_{p,m}^{{}^{\circ}}(g)$ of the gaseous compound and the constant pressure standard molar heat capacity $C_{p,m}^{{}^{\circ}}(cr)$ of the crystalline compound. The value is the arbitrary reference temperature *θ* = 298.15 K; *p*^0^ = 10^5^ Pa is the reference pressure. The obtained results are presented in Table 1.

IVZ has quite high standard molar enthalpy of sublimation (138.1 kJ·mol^−1^), which is associated with the presence of two aromatic rings and two heterocycles with a big molecular weight in the compound structure. Additionally, the N, F, and S atoms of the heterocycles as H-acceptors are capable of specific interactions through the formation of an intermolecular hydrogen bond with the H-donor hydroxyl group of the neighboring molecules. It can be assumed that additional structuring of the crystalline phase is possible due to the coplanarity and π-π stacking of the cyclic fragments of the compound molecules, which also makes the crystal lattice energy higher.

#### 2.2.2. Clusterization Approach

The experimental data were analyzed by means of a database created by us [18], which includes experimental material published in the literature. The database accumulates the various information: the experimental method; the temperature interval for measurements; the sublimation Gibbs energy and enthalpy at 298.15 K; the melting temperatures and fusion enthalpies of the selected molecular crystals and the ref codes [19] of the compounds which have been evaluated by single crystal X-ray diffraction experiments. Sometimes the database involves enthalpies and Gibbs energies received by different methods and temperatures; in this case it was applied an algorithm for reducing these values to comparable conditions. If the same compound had been evaluated before in the literature by several approaches, preference was given to the method which obtained sublimation Gibbs energy (saturated vapor pressure) and enthalpy data at the same time. It should be noted that, in the case if the other conditions being identical, we used the data with the temperatures maximally close to the standard condition (298.15 K). The temperature dependence was used for evaluation of the saturated vapor pressure at 298.15 K.

The Tanimoto similarity indices (*T_c_*) were used for estimation of the structural similarity of the compounds selected by means of the MOLDIVS (MOLecular DIVersity & Similarity) program [20]

*T_c_* = *N*(*A*&*B*)/[*N*(*A*) + *N*(*B*) − *N*(*A*&*B*)] (2)

where *N*(*A*) is the number of fragments in molecule *A*, *N*(*B*) is the number of fragments in molecule *B*, *N*(*A*&*B*) is the number of common fragments in molecules *A* and *B*.

Molecular fragments are determined as atom-centered concentric environments. The fragments include a central atom and neighboring atoms attached with it within a predefined sphere size (the number of bonds between the central and edge atoms). The parameters such as bond type, charge, valency, cycle type and size of the atom were coded by fixed-length variables. The (MOLecular DIVersity & Similarity) program evaluates the similarity of each molecule in the database with all the other molecules arranged by likeness with the initial molecule.

The descriptors applied were obtained by the program package HYBOT-PLUS (version of 2003) in Windows [21].

The special algorithm was used for evaluation of the interaction peculiarities between the molecules in the crystal lattice. The substances of the database were dissected into structurally similar groups/clusters. Within each group we have similar parameters describing these crystal structures. The similarity of characteristics should improve statistical values of the correlation equations. We selected the procedure for producing fragmentation of the database including groups/clusters with structurally similar compounds. The Tanimoto similarity coefficients *T_c_* (*T_c_* = 0: no similarity; *T_c_* = 1: identity) were applied for creation of the groups/clusters. For compound selection belonging to the same cluster, we used a criterion: 0.75 ≤ *T_c_* ≤ 1. As a result, 17 compounds with experimental Gibbs energies and enthalpies of sublimation were selected (Table S1). Within the formed cluster for IVZ, we tried to find a correlation between the Gibbs energies of sublimation $\Delta_{cr}^{g}G_{m}^{{}^{\circ}}(298.15\;K)$ and the physicochemical descriptors of HYBOT. Using the entire set of descriptors, the best correlations were observed for molecular polarizability (α) (Figure S1a) and total acceptor ability of a molecule to form hydrogen bonds (∑(*C*_*a*_)) (Figure S1b). As a result, a two-parameter correlation equation was obtained to estimate $\Delta_{cr}^{g}G_{m}^{{}^{\circ}}(298.15\;K)$:

(3) $$\Delta_{cr}^{g}G_{m}^{{}^{\circ}}(298.15\;K)=(5.00\pm 3.35)+(0.955\pm 0.229)\cdot \alpha +(3.571\pm 0.806)\cdot \Sigma (C_{a})$$

R = 0.9347; SD = 4.81 kJ·mol^−1^; *n* = 17; F = 48.44.

In order to obtain a complete thermodynamic picture of IVZ sublimation process, it was necessary to estimate the value of enthalpy. For this purpose, we used the correlation dependence between the Gibbs energies and the enthalpies of sublimation (the so-called “compensation effect”) within the selected cluster (Figure S2). As a result, the following equation was obtained:

(4) $$\Delta_{cr}^{g}H_{m}^{{}^{\circ}}(298.15\;K)=(50.8\pm 2.6)+(1.197\pm 0.077)\cdot \Delta_{cr}^{g}G_{m}^{{}^{\circ}}(298.15\;K)$$

R = 0.9707; SD = 3.87 kJ·mol^−1^; *n* = 17.

The sublimation thermodynamic functions of IVZ calculated from these equations are in good agreement with the experimental values: $\Delta_{cr}^{g}G_{m}^{{}^{\circ}}{\left(298.15\;K\right)}_{cal}$ = 76.2 kJ·mol^−1^ and $\Delta_{cr}^{g}G_{m}^{{}^{\circ}}{\left(298.15\;K\right)}_{\exp}$= 68.9 ± 0.2 kJ·mol^−1^; $\Delta_{cr}^{g}H_{m}^{{}^{\circ}}{\left(298.15\;K\right)}_{cal}$ = 142.0 kJ·mol^−1^ and $\Delta_{cr}^{g}H_{m}^{{}^{\circ}}{\left(298.15\;K\right)}_{\exp}$ = 138.1 ± 0.5 kJ·mol^−1^.

The analogous analysis for estimation of the sublimation thermodynamic functions of isavuconazole with applying database clusterization is presented for bicalutamide (as an ancestor of the cluster) in Supplementary Material (Table S2, Figures S3 and S4).

### 2.3. Solubility

To describe the behavior of IVZ in model biological media, we measured its solubility in acidic (pH 1.2 and 2.0) and weakly alkaline (pH 7.4) buffer solutions modeling the gastric acid medium and blood plasma, respectively. Additionally, the data on solubility were obtained in ethanol, 1-propanol, 1-octanol and hexane that are considered to be pharmacologically relevant solvents and are widely used to produce and purify drug compounds. Ethanol, the molecule of which consists of both hydrophobic and hydrophilic parts, is a solvent that is often used as a cosolvent and a preservative and takes part in pharmaceutical preparation transport in the body. It is useful to determine the solubility value in amphiphilic 1-octanol to estimate the ability of a substance to permeate through lipophilic membranes [22]. Hexane, which is only capable of nonspecific van der Waals interactions with solute molecules, helps to identify the role of hydrogen bonding in the dissolution process.

**Figure 4:**
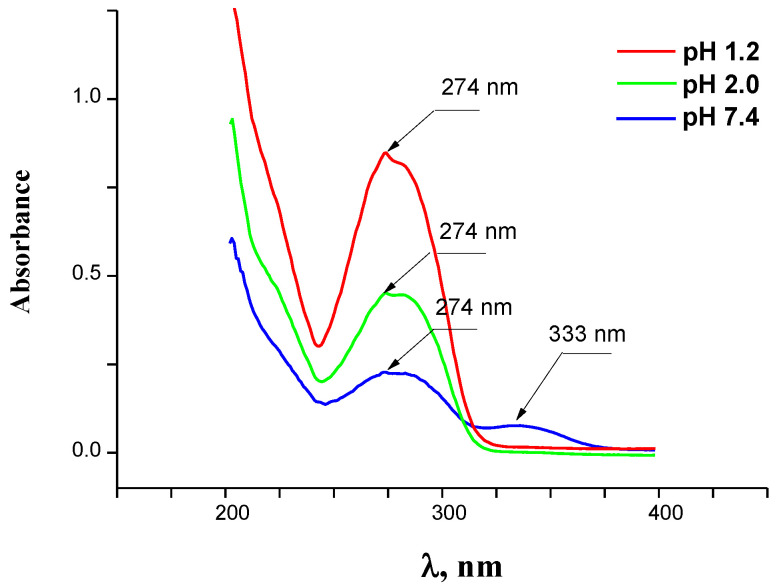
UV–visible absorption spectra of IVZ in buffers solutions (pHs 1.2, 2.0 and 7.4).

The IVZ absorption spectra in buffer solutions (pH 1.2, 2.0 and 7.4) were obtained within the range from 200 to 400 nm and are shown in Figure 4. The UV spectra of the compound aqueous solutions at all the pH values have an intensive absorption band with a maximum at 274 nm. As the acidity becomes lower, a new absorption band is formed around 333 nm. The adsorption maxima at 274 and 333 nm in the buffer solutions correspond to π-π* and *n*-π* transitions in the aromatic parts of isavuconazole. The changes in the UV spectra at various pH values are caused by ionization of the molecules of the compound under study.

**Figure 5:**
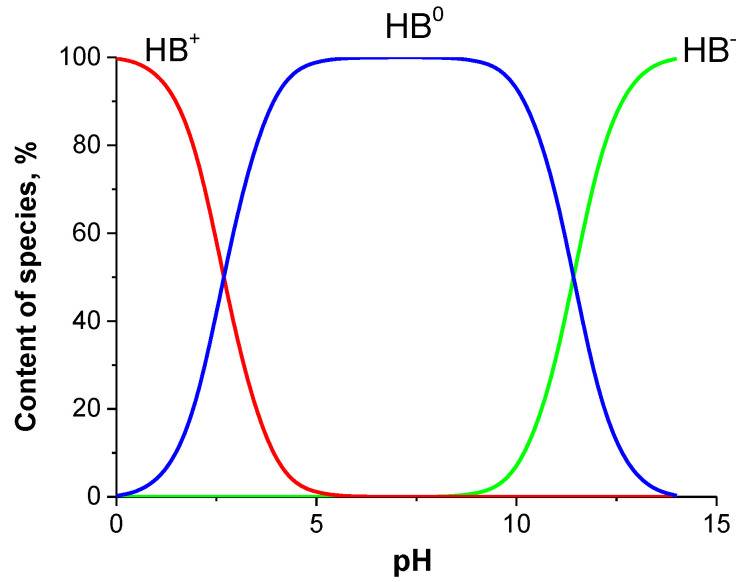
Distribution of species as a function of pH of buffer solution for IVZ.

The IVZ solubility values in buffer solutions of different acidity, ethanol, propanol, 1-octanol and hexane within the temperature range of 293.15–313.15 K were determined by the isothermal saturation method and are presented in Table 2 and Figure 5. After the solubility experiments, the bottom phases were removed from the saturated solutions and analyzed by the PXRD method. The results in Figure S5 (Supporting Information) show that IVZ was stable during the solubility experiments in all the studied solvents and there were no solvated forms.

The IVZ solubility in aqueous buffer solutions at 298.15 K changed within the range of (8.13–42.40) × 10^−6^ mol L^−1^ and increased in the following order: buffer pH 7.4 ˂ buffer pH 2.0 ˂ buffer pH 1.2. The solubility value in the buffer pH 7.4 was 80% lower than that in the pH 1.2 one. The differences between the solubility values of the drug compound in the buffer solutions used are explained by the protolytic properties of the compound under study. Molecules of most drugs are ionized in an aqueous solution as they contain at least one acidic or basic functional group. That is why such molecules can exist in the neutral (uncharged) or ionized (charged) forms, depending on the solution pH [23]. The isavuconazole molecule contains eight acceptor centers (basic nitrogen, sulfur and fluorine atoms) and one donor group (acidic hydroxy group). Depending on the medium pH value, IVZ molecules in aqueous solutions are found in three forms: neutral nonionized form BH^0^, responsible for diffusion through biomembranes, and two ionized ones—in the form of a positively charged BH^+^ cation and a negatively charged BH^−^ anion. The pKa values of isavuconazole were calculated using Advanced Chemistry Development (ACD/Labs) Software V11.02 and equaled: pKa_1_ = 2.70 ± 0.10 (most basic); pKa_2_ = 11.42 ± 0.29 (most acidic). The scheme of IVZ protolytic equilibria can be represented as follows:

$$HB^{+}\overset{pKa1_{a1}}{↔}HB^{0}\overset{pKa2}{↔}HB^{-}$$

The Henderson–Hasselbalch equation and dissociation constants were used to determine the content of molecular and ionized forms of isavuconazole molecules at different pH values of buffer solutions (Figure 5) [24].

The acid–base equilibrium diagram indicates that the buffer pH 1.2 contains approximately 7% neutral forms and 93% cationic forms of the drug molecules. When the buffer acidity decreases to pH 2.0, the content of the neutral molecules goes up to 22%, whereas that of ionized particles becomes lower and makes up 78%. All the drug molecules in the weak alkaline buffer pH 7.4 are in the neutral molecular form. The presence of ionized forms of IVZ molecules in the solutions explains the higher solubility in the buffers pH 1.2 and 2.0 in comparison with the pH 7.4 one.

**Figure 6:**
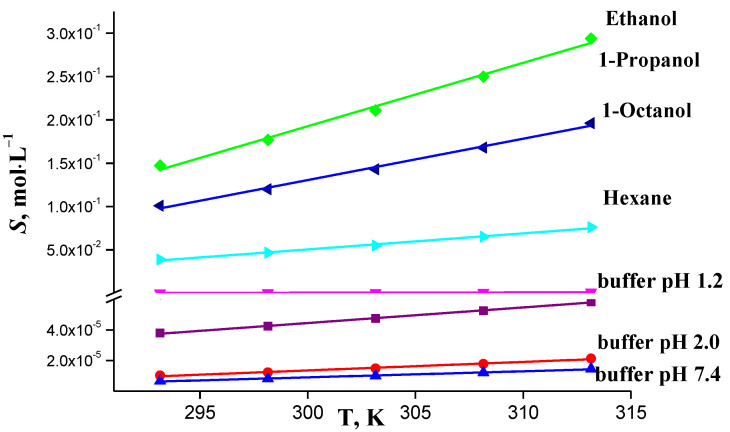
Temperature dependences of IVZ solubility in selected solvents.

The solubility of the drug compound increases with the temperature in all the studied solvents, as the experimental data show (Figure 6). The biggest temperature gradient of solubility is found in hexane. The isavuconazole solubility in hexane is higher than in buffer solutions, which is explained by the dispersion interactions of nonpolar aromatic fragments of the IVZ molecule with the hexane nonpolar molecules. The isavuconazole solubility in alcohols is much higher than in hexane and buffer solutions, which is associated with the formation of hydrogen bonds during the interaction of proton acceptors of the aromatic and heterocyclic systems of the dissolved drug with the hydroxy group of the alcohol molecules. The IVZ solubility increase in the series of alcohols from 1-octanol to ethanol is explained by the growing solvent capacity for specific interactions [25].

### 2.4. Hansen Solubility Parameters

The HSP parameters of IVZ and the selected solvents, as well as the calculated molar volumes at 298.15 K, are summarized in Table 3. The group contribution parameters for the respective molecular forces and the associated molar volumes with the number of the corresponding group for the compound studied are listed in Table S3. The calculation results showed that the Δ*δ* values of IVZ in the used organic solvents are lower than in buffer solutions, which corresponds to the measured values of solubility. However, a comparative analysis of Δ*δ* for alkanols did not show the experimentally observed increase in the compatibility of the drug with alcohols with a decrease in their alkyl radical. In this case, the contributions of the parameters *δ_d_* and *δ_p_* of alcohols to the value of Δ*δ* are approximately equal, and the resulting difference in the results is introduced by the parameter *δ_h_*, which reflects the ability of solvents to form hydrogen bonds.

**Figure 7:**
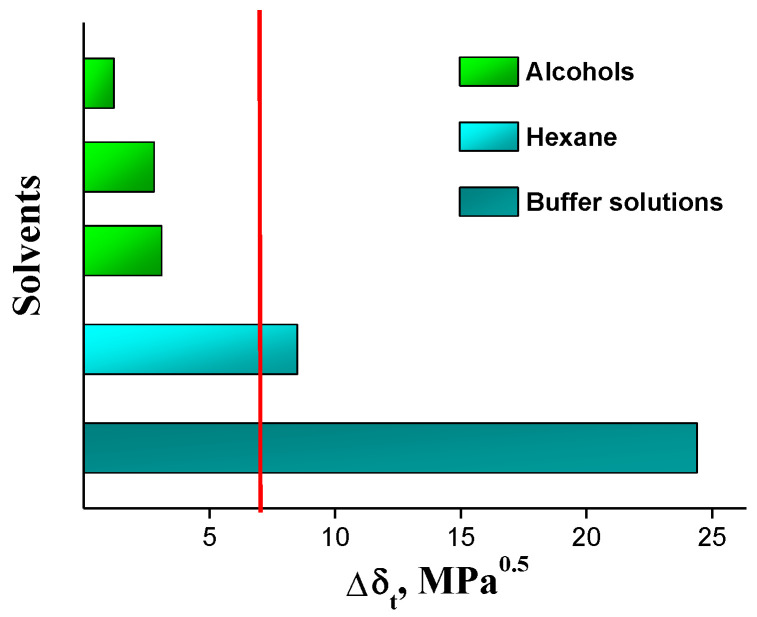
Differences in the total HSPs of IVZ with solvents. The red line is an indicator of miscibility.

The parameter ∆*δ_t_* can be used as a criterion for the miscibility of a solute with a solvent [26]. Solvents located at a distance <10 MPa^0.5^ from the solute are considered good solvents for this compound and those solvents that fall outside these limits are considered nonsolvents. The solutions with ∆*δ_t_* in the range of 7–10 MPa^0.5^ were partially miscible. The presented diagram for IVZ indicates that ethanol, 1-propanol and 1-octanol will exhibit good solubility, while buffer solutions will potentially show themselves as the weakest solvents (Figure 7). The value ∆*δ_t_* in hexane is 8.5, which corresponds to the average miscibility. This result provides a good approximation of the IVZ behavior in the investigated solvents with the measured solubility data.

### 2.5. Solubility Data Modeling

The most common thermodynamic calculation models—the Apelblat and van’t Hoff ones—were used to model isavuconazole solubility in the seven solvents selected [27,28]. We employed the relative deviation (*RD*), relative average deviation (*RAD*) and root mean square deviation (*RMSD*) between the experimental and calculated solubility values of the compound studied to assess the applicability and to verify the correlation results of these models. The experimental solubility values in the studied solvents expressed in molar fraction and IVZ solubility values calculated are shown in Table 4. The modeling parameters and experimental deviation values are given in Table 5.

An analysis of the data in Table 4 and Table 5 allows us to conclude that the experimental data and solubility values calculated by the Apelblat and van’t Hoff equations agree well with each other. Both models can be considered suitable for solubility correlation in all the studied solvents. As Table 4 shows, the relative deviations (10^3^*RD*) increase in the following order: ethanol (0.48) < hexane (0.58) < buffer pH 1.2 (1.26) < propanol (2.58) < 1-octanol (2.66) < buffer pH 2.0 (3.94) < buffer pH 7.4 (3.98) for the modified Apelblat model, whereas for the van’t Hoff model: hexane (0.62) < propanol (1.08) < buffer pH 1.2 (1.72) < ethanol (2.46) < 1-octanol (3.18) < buffer pH 7.4 (4.28) < buffer pH 2.0 (5.74). As the calculation results in Table 5 show, the employed thermodynamic models allow determining the solubility values with the *ARD* less than 1%. Based on the total average *RD*, *ARD* and *RMSD* values, we established that the modified Apelblat model has better agreement with the experimental data on the compound solubility in the selected solvents.

### 2.6. Dissolution Thermodynamics

The calculated values of *x*_id_ and activity coefficient (ln*γ^∞^*) for isavuconazole at infinite dilution in the studied solvents are shown in Table 6.

**Figure 8:**
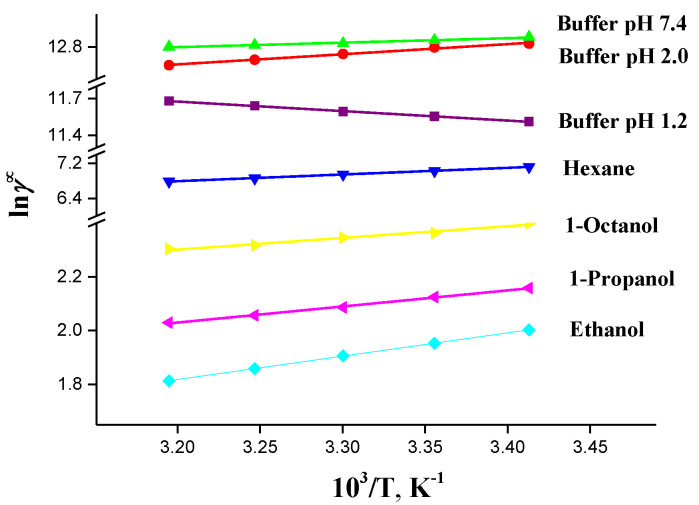
Temperature dependencies of activity coefficients at infinite dilution of IVZ in studied solvents.

In all the solute–solvent systems, positive deviation from ideality (*γ^∞^ >* 1) was observed, which indicates a weak intermolecular interaction of IVZ when aqueous and organic solvents are used. An analysis of the data obtained shows that the drug activity coefficients in the buffer solutions (pH 2.0 and 7.4) and organic solvents become lower as the temperatures increase, which is associated with the solute–solvent interactions and solubility growth. In the buffer solution (pH 1.2), the drug activity coefficients become slightly higher as the temperature grows (Figure 8).

The IVZ activity coefficients decrease in the following order, depending on the solvent chemical nature: buffer pH 7.4 > buffer pH 2.0 > buffer pH 1.2 > hexane > 1-octanol >1-propanol > ethanol, which agrees with the order of the drug solubility growth in these solvents (Table 2).

The IVZ excess partial thermodynamic functions were calculated based on the linear dependences of ln*γ^∞^* on reciprocal temperature (Figure 8), with the results summarized in Table 7.

**Figure 9:**
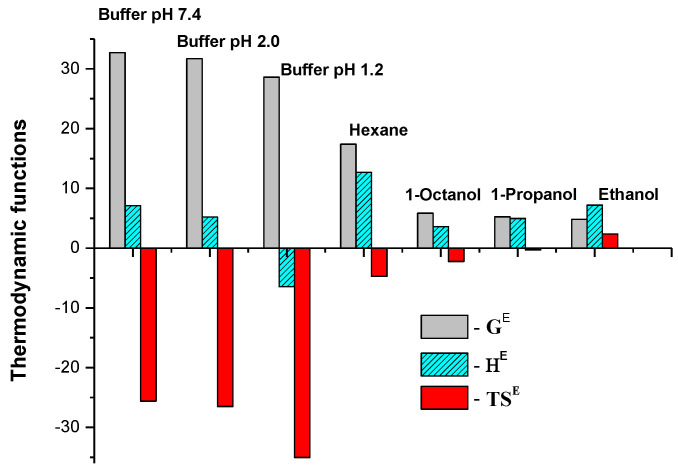
Excess thermodynamic solubility functions of IVZ in the investigated solvents.

For the best visualization, the excess thermodynamic solubility functions calculated from the van’t Hoff plot are illustrated as a diagram (Figure 9). In all the binary isavuconazole-solvent systems, the Gibbs energy values were positive (*G^E^* > 0), which indicates that the dissolution process was hindered. It was established that the entropy term of the Gibbs energy in aqueous solutions exceeds the enthalpy one *H^E^ ˂ TS^E^* (in absolute value), which indicates that the entropy is the main cause of the deviation from ideality in these solutions. In case of organic solvents, the main contribution to the deviation from ideality is the enthalpy term of the Gibbs energy: *H^E^ ˃ TS^E^* (in absolute value).

The dissolution Gibbs energy values of IVZ in the alcohols are lower than in hexane, according to the solubility data, which is associated with the more positive enthalpy values, *H^E^*, in the alkane than in the alkanols. In the alcohol series, the longer alkyl chain makes the deviation from ideality bigger. The entropy term in the ethanol solution *TS^E^ ˃* 0, which is favorable for the thermodynamic stability of the solution and ensures the highest solubility in this alcohol.

The enthalpy and entropy contributions shown in Table 7 indicate that in the systems with hexane and alcohols, the entropy term is lower (ζ_TS_^E^ < 32%) than in the buffer solutions, where this contribution is the factor that determines the solubility (ζ_TS_^E^ > 78%).

### 2.7. Lipophilicity

Lipophilicity is a physicochemical property of compounds that make the biggest contribution to their membrane permeability. The lipophilicity measure is the compound partition coefficient in the 1-octanol/water system that represents the logarithm of the ratio of concentrations of a nonionized substance in the system of two immiscible liquids (log*P*) [29].

When studying the protolytic properties (Figure 5), we established that the isavuconazole molecules in the buffer pH 7.4 were in the neutral form. That is why the obtained experimental partition coefficients of the drug compound in the 1-octanol/buffer pH 7.4 system represent the ratio of the concentrations of nonionized molecules. Table 8 shows the experimental equilibrium concentrations of IVZ in the solvents used and partition coefficients of the compound in the 1-octanol/buffer pH 7.4 system within the temperature range from 298.15 to 313.15 K.

It was established that the IVZ solubility in both phases of the binary system changed with the temperature growth in such a way that the ratio of the compound concentrations in the octanol and aqueous phases increased and, consequently, led to higher partition constant values. The data obtained show that the temperature growth makes the substance distribution in 1-octanol, imitating the membrane lipid layer, higher. The experiment showed that the isavuconazole log*P_O/B_* value at the temperature of 298.15 K was equal to 2.56, which indicates the compound under study has average lipophilicity. It should be said that the optimal interval of drug lipophilicity determined empirically is expressed by the inequality: −0.5 < log*P* < 3 [30].

The temperature dependences of the partition coefficients were used to calculate the thermodynamic functions of transfer characterizing isavuconazole transition from the buffer pH 7.4 to 1-octanol (Table 8). The value of the Gibbs energy of IVZ transfer is negative and, consequently, the compound transition from the buffer solution to the organic medium is favorable. The transfer enthalpy change is positive, and the process is endothermic. The thermal effect of compound distribution indicates that the energy of the solute–solvent interaction in the octanol phase is lower than the energy required for the breakage of intermolecular bonds in the aqueous medium. Such characteristics of intermolecular interactions are associated with hydrophobic hydration of phenyl and alkyl fragments of IVZ molecules. The entropy changes are also positive, which means that the isavuconazole molecules are more mobile and the system is less ordered during the distribution of the studied substance. Since the entropy term of the Gibbs energy of transfer is higher than the enthalpy one, the IVZ distribution is entropy-driven.

## 3. Materials and Methods

### 3.1. Materials

Details of IVZ and selected solvents for this studied are presented in Table 9. Buffer solutions were made using bidistilled water (with electrical conductivity 2.1 μS cm^−1^). Solutions of salts KHPO_4_ (9.1 g in 1 L) and NaH_2_PO_4_·12H_2_O (23.6 g in 1 L) were mixed to obtain phosphate buffer pH 7.4 (I = 0.15 mol·L^−1^). To prepare the buffer pH 2.0 (I = 0.10 mol·L^−1^), 6.57 g of KCl was dissolved in water, 119.0 mL of 0.1 M hydrochloric acid was added and the volume of the solution was adjusted to 1 L with water. By combining 425 mL 0.1 M HCl in 500 mL and 3.73 g KCl in 250 mL of water buffer solution pH 1.2 was made. The pH values of solutions were determined using a pH meter FG2-Kit (Mettler Toledo, Switzerland) calibrated by commercial standard buffers pH 1.68, 6.86 and 9.22 solutions.

### 3.2. Differential Scanning Calorimetry

The thermal behavior of the compound studied including melting temperature and enthalpies of fusion has been studied a Per-kin-Elmer Pyris 1 DSC differential scanning calorimeter (Perkin-Elmer Analytical In-struments, Norwalk, CT, USA) with Pyris software for Windows NT. DSC runs were performed in an atmosphere of flowing 20 cm^3^·min^−1^ dry helium gas of high purity 0.99996 (mass fraction) using standard aluminum sample pans and a heating rate of 2 K·min^−1^. The accuracy of weight measurements was 0.005 mg.

The equipment was calibrated using a two-point calibration with indium and zinc standards. Onset melting temperature independent of the scan rate was used for calibration. The fusion temperature for indium and zinc were 429.7 ± 0.2 °C and 692.6 ± 0.2 °C, respectively (determined by at least ten measurements). The obtained values exactly match with recommendation [31]. The enthalpy scale was calibrated using the heat of fusion of indium. The measured fusion enthalpy value equaled 28.69 ± 0.2 J.g^−1^ (its reference value is 28.66 ± 0.5 J·g^−1^ [32]. The DSC measurements were repeated in triplicate and values agreed within the experimental uncertainties *u(*∆*H_m_)* = 0.5 kJ mol 1 for the enthalpy of fusion and *u(T_m_)* = 0.2 K for the melting temperature. Uncertainties for melting parameters expanded uncertainty of the mean (0.95 confidence level).

### 3.3. Powder X-ray Diffraction

After the solubility study was complete, PXRD analysis was performed on isavuconazole powders recovered from solvents. All samples were measured at 40 kV and 40 mA using a Bruker D8 Advance diffractometer with CuKa radiation (λ = 1.5406 Å) at room temperature. Diffraction patterns for the samples were collected by changing the angle of diffraction (2*θ*) from 5° to 30° with a step size 0.03°.

### 3.4. Vapor Pressure Measurements

The transpiration method was used for the sublimation experiment. A stream of an inert gas (nitrogen) was passed over a sample at the constant temperature and flow rate; saturation of the gas with the substance vapor was achieved by low transpiration rate of gas. Then, the sublimated quantity of condensed vapor was determined. The volume of the inert gas and the amount of the sublimated material were used for calculation the vapor pressure over the sample of compound at this temperature. The following procedure was used for determination the amount of sublimed substance. The condensed sample was dissolved in a known volume of solvent *V_sol_*. The mass of the substance was determined by the measurement of absorbance A of its solution (spectrophotometer Cary-50, Varian, Palo Alto, CA, USA, Software Version 3.00 (339)). According to the Lambert–Beer law the concentration of the solution c (mol·L^−1^) can express knowing a value of the extinction coefficient ε (l·mol^−1^·cm^−1^) of the studied compound dissolved in the solvent and an absorbing path length (*l*):

*A* = *εcl* (5)

where *l* is an absorbing path length. Calculation the mass of sublimed compound was performed according to the equation:

*m* = *cV_sol_M* (6)

*M* is the molar mass of the studied substance.

The vapor pressure *p* at each temperature was determined from the amount of the substance collected within a known time:

*p* = *mRTa/VM* (7)

where *V* is the volume of the gaseous nitrogen evaluated from the time measurement and the flow rate at the flow meter temperature *Ta* and the atmospheric pressure. Uncertainties for transpiration experiment parameters (*T*, *m*, *Ta*, *V*) are reported as standard deviations (Table 1). The combined standard uncertainty of vapor pressure measurements were estimated taking into account uncertainties of all variables involved in eq 7.

The details of equipment and experimental procedure are given in [33]. The sublimation technique was tested with benzoic acid as standard [34]. The experimental vapor pressure values were obtained in the temperature interval of (307–385) K and were in good agreement with the results of studies [35,36,37] within the limits of experimental error (Figure S7). The standard sublimation enthalpy of benzoic acid measured by us was 90.5 ± 0.3 kJ·mol^−1^ and consistent with recommended by IUPAC value—89.7 ± 0.5 kJ·mol^−1^ [38]. Each experiment at fixed temperature was repeated three times with the standard deviation of up to 5%.

### 3.5. Solubility

Solubility of drug was measured by the classical shake flask method at five temperatures: 293, 298, 303, 308 and 313 ± 0.2 K. This method determines the concentration of a test substance in a saturated solution, i.e., in equilibrium with the solid phase at a strictly fixed temperature. The compound studied and the selected solvent were placed into glass ampoules and stirred in an air thermostat. The time to reach equilibrium in the solvent–solute system was determined from the kinetic dependences of solubility and was 20–24 h for all investigated solutions.

The solid phase was sedimentated after stirring within 2 h. Then, the solutions were centrifuged using a thermostatic centrifuge Biofuge Stratos (Thermo scientific, Germany) at the appropriate temperature of the experiment for 5 min. The solid phase was removed by filtration using a filter MILLEX^®^HA 0.22 μm (Millipore, Ireland). The resulting solution, if necessary, was diluted with an appropriate solvent and examined on a Carry 50 spectrophotometer (Varian, Palo Alto, CA, USA, Software Version 3.00 (339)) in the ultraviolet region of the spectrum (operating wavelength range λ = 200–400 nm) with an accuracy of 2–4%.

The experimental value of solubility is an average value of three independent experiments. The absorption maximums for isavuconazole in the selected solvents have been determined at 274 nm in buffers solutions (Figure 4) and as 283 nm in hexane and alcohols (Figure S8). The calibration was carried out at room temperature using the solutions with known concentrations of drug in selected solvents.

The mole fraction concentration was calculated based on molarity (*S*, mol·L^−1^) using Equation (8):

(8) $$x=\frac{M_{2}S}{S(M_{2}-M_{1})+1000\rho },$$

where *M*_1_ and *M*_2_ are the molar masses of solute and solvent, respectively, and *ρ* (g·cm^−3^) is the density of the pure solvents. Densities of buffers solutions (pH 1.2, 2.0 and 7.4) were measured using densitometer DMA 4500 (Anton Paar, Austria) and published earlier [39]. The values of densities using for conversion are given in Table S4 (Supporting Information).

### 3.6. Partition Experiment

The partition coefficients in the 1-octanol/buffer pH 7.4 system were determined by the shake flask method in the temperature range 293.15–313.15 K. The total volume of the two-phase system 80 cm^3^ with the ratio of the organic phase to the volume of the aqueous phase 1:1 was used. Before the experiment, both solvents were mutually saturated by stirring in a large vessel for two days. The stock solution with the test compound was prepared in a solvent that demonstrated the best solubility. The flasks with the test compound were stirred for 24 h in an air thermostat until complete equilibrium was ensured, as evidenced by the absence of turbidity in each of the phases. The final drug concentrations in both immiscible phases were measured spectrophotometrically.

The partition coefficients in 1-octanol/buffer system (*P_O_*_/*B*_) were calculated as the ratio of the equilibrium molar concentrations in the organic (*s_O_*) and buffer (*s_B_* )phases:

*P_O/B_ =s_O_/s_B_* (9)

The value P*_O/B_ expressed in mole fraction were determined by follow equation:

P*_O/B_ = x_O_/x_B_ (10)

The standard transfer enthalpy of drug was calculated based on the experimental temperature dependence of partition coefficients using van’t Hoff method:

(11) $$\frac{d(\ln P_{O/B}^{*})}{dT}=\frac{\Delta_{tr}H^{0}}{RT^{2}}$$

whereas the standard Gibbs energy of transfer process from the buffer to 1-octanol was evaluated:

∆_tr_*G*° = −RTlnP*_O__/*B*_ (12)

The standard transfer entropy (∆_tr_*S*°) is obtained by means of relationship:

∆_tr_*S*° =(∆_tr_*H*° − ∆_tr_*G*°)/*T* (13)

The transfer thermodynamic functions represent the change in the enthalpy and entropy terms when one solute mole is transferred from the aqueous phase to the organic phase at infinite dilution.

### 3.7. Theoretical Basis

#### 3.7.1. Hansen Solubility Parameters

The Hansen solubility parameters (HSPs) are physicochemical parameters and are widely used to estimate the type of interactive forces responsible for compatibility between materials [40]. Hansen proposed that the cohesive energy density of a solvent results from the summation of energies of volatilization from all of the intermolecular attractions present in the liquid:

Δ*E_t_/V_m_* = Δ*E_d_/V_m_* + Δ*E_p_/V_m_* + Δ*E_h_/V_m_,* (14)

where Δ*E*, subscripts *t*, *d*, *p,* and *h,* respectively, represent the energies per mole of solvent, and energy contributions arising from dispersion, polar, and hydrogen bonding, respectively. *V_m_* is the molar volume. Alternatively, this may be written in terms of the solubility parameter *δ_t_*, in the form:

*δ_t_*^2^ =
*δ_d_*^2^ +
*δ_p_*^2^ +
*δ_h_*^2^ (15)

where *δ_t_, δ_d_, δ_p_* and *δ_h_* are the solubility parameters corresponding to the solvent, dispersion, polar and hydrogen bonding, respectively.

The difference between the solubility parameter of solute and solvent could then be determined using the ∆*δ_t_* and ∆*δ* factors [41]:

(16) $$\Delta \delta_{t}=\delta_{t1}-\delta_{t2}$$

(17) $$\Delta \delta ={\left({\left(\delta_{d1}-\delta_{d2}\right)}^{2}+{\left(\delta_{p1}-\delta_{p2}\right)}^{2}+{\left(\delta_{h1}-\delta_{h2}\right)}^{2}\right)}^{0.5}$$

The HSP of a substance is often calculated by group contribution methods, in which the only datum required for the calculation is the compound’s chemical structure [42,43,44]. In this study, we use improved group contribution parameters to calculate the HSP. The parameters were developed based only on the data about the pharmaceutical solids suggested by Just et al. [45]. The solubility components (*δ_d_, δ_p_*, *δ_h_*) were calculated by the following equations:

*δ_d_* = Σ*F_di_*/Σ*V_i_* (18)

*δ_p_* = (Σ*F_pi_*^2^)^0.5^/Σ*V_i_* (19)

*δ_h_* = (Σ*F_hi_*/Σ*V_i_*)^0.5^ (20)

where *V_i_*, *F_di_*, *F_pi_* and *F_hi_* illustrate the contributions to molar volume, the dispersion force, polar force and hydrogen bonding energy for group *i*.

#### 3.7.2. Modified Apelblat Model

The modified Apelblat model [46] developed for nonideal solutions is one of the most frequently used models for prediction and correlation of solubility data and can be expressed as follows:

(21) $$\ln x=A+\frac{B}{T/K}+C\ln(T/K)$$

where *x* is the compound solubility in the studied solvents, which can be determined by Equation (8); T is the absolute temperature; *A*, *B* and *C* are three empirical constants of the equation. The *A* and *B* values represent changes in the solution behavior as a result of the nonideality of the solute solubility, whereas *C* reflects the connection between the melting point and enthalpy [47]. Despite its simple mathematical expression, this model can ensure high accuracy of prediction of the solute solubility at a variety of temperature values [48].

#### 3.7.3. Van’t Hoff Equation

The van’t Hoff equation can be used to calculate the relationship between the solubility of solute and temperature [49]. The equation can be simplified as Equation (22).

(22) $$\ln x=A+\frac{B}{\left(T/K\right)}$$

where *x* represents the mole fraction solubility of compound. *T* is the absolute temperature and *A, B* are the regression parameters obtained using multivariable least-square method [50].

#### 3.7.4. Data Correlation

The relative deviations (*RD*) were calculated according to

*RD = (x_exp_ − x_cal_)/x_exp_* (23)

The relative average deviations (*RAD*) were evaluated by equation:

(24) $$RAD=\frac{1}{N}\sum_{i=1}^{N}{\left|\frac{x_{\exp}-x_{cal}}{x_{\exp}}\right|}^{}$$

The root-mean-square deviation (*RMSD*) are defined as:

(25) $$RMSD={\left|\frac{1}{N}\sum_{i=1}^{N}{\left(x_{\exp}^{}-x_{cal}^{}\right)}^{2}\right|}^{1/2}$$

where *N* represents the number of experimental points and *x*_exp_ and *x_cal_* are the experimental and calculated mole fraction solubility values of the compound, respectively.

### 3.8. Dissolution Thermodynamics

The temperature dependence of solute solubility is described by the thermodynamic relationship [51]:

(26) $$\ln x=\ln x_{}^{id}-\ln\gamma_{}=\frac{\Delta_{m}H}{RT}\left[\frac{T-T_{m}}{T}\right]+\overset{T}{\underset{T_{m}}{\int }}\frac{\overset{T}{\underset{T_{m}}{\int }}(C_{p}^{L}-C_{p}^{S})dT}{RT^{2}}-\ln\gamma_{}$$

where *x*, *x*_id_, *γ*, *T_m_*, Δ*_m_H*, $\Delta C_{p}=C_{p}^{L}-C_{p}^{S}$, *R*, and *T* represent the solubility mole fraction of the solute in solution, ideal mole fraction solubility of the solute, activity coefficient of the solute in solution, melting temperature of the solute, enthalpy of melting of the pure solute, differential molar heat capacity of the pure solute, gas constant and temperature, respectively. If the solubility or equilibrium mole fraction of a compound in solvents studied is low, it is assumed that the last term in Equation (26) denotes the infinite dilution activity coefficient ln*γ*^∞^, which can be expressed as:

(27) $$\ln\gamma_{}^{\infty }=\frac{\Delta H_{sol}^{E}}{RT}-\frac{\Delta S_{sol}^{E}}{R}$$

where $\Delta H_{sol}^{E}$ and $\Delta S_{sol}^{E}$ represent the partial molar excess enthalpy, and partial molar excess entropy, respectively, and are assumed to be temperature independent. As the quantity Δ*C_p_* is assumed to be negligible and considered to be zero [52], substituting Δ*C_p_* = 0 into Equation (26) simplifies to

(28) $$\ln x_{id}^{}=-\frac{\Delta H_{m}}{RT_{m}}\ln\left[\frac{T_{m}}{T}\right]$$

## 4. Conclusions

New experimental data for physico-chemical properties of the antimycotic drug isavuconazole in solid state and solutions were obtained. The thermophysical parameters of the compound were determined: the temperature and enthalpy of melting are 302.7 K and 29.9 kJ mol^−1^, respectively. The vapor pressure of IVZ was measured by the transpiration method in the temperature range 365.15–383.15 K and the sublimation thermodynamic functions were calculated by the Clark–Glew equation. The standard sublimation enthalpy of the compound is 138.1 kJ mol^−1^. Using the shake-flask method, the solubility of IVZ in seven pharmaceutically significant solvents was studied at a temperature of 293.15–313.15 K. The solubility of the drug increased in the following order: buffer pH 7.4, buffer pH 2.0, buffer pH 1.2, hexane, 1-octanol, 1-propanol, ethanol. Solubility values of IVZ at 298.15 K changed from 8.13·10^−6^ mol·L^−1^ in a buffer with a pH of 7.4 to 0.18 mol·L^−1^ in ethanol. The solubility of the uncharged forms of the IVZ molecule in the buffer pH 7.4 is five times lower than the solubility of the ionized forms in the buffer with a pH of 1.2. Hansen solubility parameters for drug and used solvents were estimated. The obtained experimental data on solubility were successfully approximated by two thermodynamic models using the van’t Hoff and modified Apelblat equations.

The activity coefficients of isavuconazole in each solvent were calculated for infinite dilution based on experimental solubility and thermophysical characteristics of the compound. The positive deviation from ideality was observed in all investigated solutions. On the basis of the temperature dependence of the activity coefficient values, the excess partial thermodynamic functions of dissolution for isavuconazole were determined.

The temperature dependences of the IVZ partition coefficients in the two-phase system 1-octanol/buffer pH 7.4 were measured, and the thermodynamic functions of the transfer process were evaluated. It was concluded that the transfer process of IVZ from the buffer solution to the organic medium is spontaneous and entropy-controlled.

## Figures and Tables

**Table 1:** Transpiration experiment parameters, vapor pressure of IVZ at different temperatures and standard sublimation thermodynamic functions.

^a^*T*/K	^b^ 10^3^ *m*/mg	^c^*V*(*N*_2_)/dm^3^	^d^*T_a_*/K	Flow/dm^3^·h^−1^	10^3^ *p*/Pa	ln*p*
365.15	3.38	9.802	297.15	1.69	1.95	−6.241
367.15	4.09	9.210	296.15	1.69	2.50	−5.991
369.15	5.16	9.092	295.15	1.69	3.19	−5.749
371.15	6.76	9.430	295.15	1.69	4.02	−5.515
372.15	7.74	9.548	295.15	1.69	4.55	−5.393
373.15	8.72	9.582	295.15	1.69	5.11	−5.277
374.15	9.35	9.160	295.15	1.69	5.72	−5.163
376.15	11.48	8.923	295.15	1.69	7.22	−4.931
378.15	14.69	9.329	295.15	1.69	8.83	−4.729
379.15	15.84	9.041	295.15	1.69	9.83	−4.622
380.15	17.09	8.568	295.15	1.69	11.19	−4.493
381.15	20.21	9.244	296.15	1.69	12.30	−4.398
382.15	20.92	8.534	296.15	1.69	13.79	−4.283
383.15	23.59	8.636	296.15	1.60	15.37	−4.175
$\Delta_{cr}^{g}G_{m}^{{}^{\circ}}(298.15\;K)$/kJ·mol^−1^				68.9 ± 0.2		
$\Delta_{cr}^{g}H_{m}^{{}^{\circ}}(298.15\;K)$/kJ·mol^−1^				138.1 ± 0.5		
$\Delta_{cr}^{g}C_{p,m}^{{}^{\circ}}$/J·mol^−1^·K^−1^				−71.8		
$\Delta_{cr}^{g}S_{m}^{{}^{\circ}}(298.15\;K)$/J·mol^−1^·K^−1^				230.4 ± 4.6		

^a^ Saturation temperature (*u*(*T*) = 0.15 K). ^b^ Mass of the transferred sample condensed (*u*(*m*) = 0.001 mg). ^c^ Volume of nitrogen used to transfer a mass of sample (*u*(*V*(*N*_2_)) = 0.003 dm^3^. ^d^ *T_a_* is the temperature of the flow rate meter used for measuring the gas flow (*u*(*T_a_*) = 0.15 K). The combined standard uncertainty for vapor pressure *u(p)/p* = 5%.

**Table 2:** Temperature dependences of solubility (*S*, mol·L^−1^) for IVZ in the selected solvents at pressure *p* = 0.1 MPa.

*T*/K	^a^ Buffer pH 1.2	^b^ Buffer pH 2.0	^c^ Buffer pH 7.4	Hexane	Ethanol	1-Propanol	1-Octanol
	(*S*·10^5^)	(*S*·10^5^)	(*S*·10^6^)	(*S*·10^4^)	(*S*·10^1^)	(*S*·10^1^)	(*S*·10^2^)
293.15	3.80	1.03	6.67	4.23	1.47	1.01	3.89
298.15	4.24	1.23	8.13	5.37	1.77	1.20	4.67
303.15	4.74	1.49	10.00	6.77	2.11	1.43	5.50
308.15	5.25	1.79	12.16	8.47	2.50	1.68	6.52
313.15	5.84	2.14	14.55	10.50	2.94	1.96	7.60

^a^ Composition of aqueous buffer pH 1.2: KCl (3.73 g in 1 L) and 0.1 mol·L^−1^ hydrochloric acid (850 mL in 1 L); ^b^ composition of aqueous buffer pH 2.0: KCl (6.57 g in 1 L) and 0.1 mol·L^−1^ hydrochloric acid (119.0 mL in 1 L); ^c^ composition of aqueous buffer pH 7.4: KH_2_PO_4_ (9.1 g in 1 L) and Na_2_HPO_4_·12H_2_O (23.6 g in 1 L). Standard uncertainties are *u*(*T*) = 0.15 K, *u*(*p*) = 3 kPa, *u*(pH) = 0.02 pH units. The relative standard uncertainties are *u_r_*(*x*) = 0.04 and *u_r_*(*S*) = 0.04.

**Table 3:** Molar volumes and Hansen solubility parameters for IVZ and selected solvents.

Compound	*V*, cm^3^·mol^−1^	*δ_d_*, MPa^0.5^	*δ_p_*, MPa^0.5^	*δ_h_*, MPa^0.5^	*δ_t_*, MPa^0.5^	*^e^* Δ*δ_t_*	*^f^* ∆*δ*	*δ_v_*, MPa^0.5^
IVZ	389.7	21.8	4.3	7.3	23.4	-	-	22.2
Buffer solutions	18.0	15.5	16.0	42.3	47.8	24.4	37.4	22.3
Hexane	131.6	14.9	0.0	0.0	14.9	8.5	10.9	14.9
Ethanol	58.5	15.8	8.8	19.4	26.5	3.1	14.2	18.1
1-Propanol	75.2	16.0	6.8	17.4	24.6	1.2	11.9	17.4
1-Octanol	157.7	17.0	5.0	11.9	20.6	2.8	6.7	17.7

**Table 4:** Experimental (*x*_exp_) and correlated (*x_cal_*) mole fractions of compound studied solubility in the solvents studied at different temperatures and pressures *p* = 0.1 MPa.

*T*/K	*^x^*_exp_	Modified Apelblat Equation		van’t Hoff Equation	
		*x_cal_*	10^3·^*RD*	*x_cal_*	10^3^ *RD*
Buffer pH 1.2 ^a^					
293.15	6.8309·10^−7^	6.8301·10^−7^	0.1228	6.8234·10^−7^	1.1027
298.15	7.6422·10^−7^	7.6469·10^−7^	−0.6118	7.6519·10^−7^	−1.2718
303.15	8.5596·10^−7^	8.5388·10^−7^	2.4392	8.5488·10^−7^	1.2713
308.15	9.4893·10^−7^	9.5109·10^−7^	−2.2742	9.5164·10^−7^	−2.8588
313.15	10.578·10^−7^	10.568·10^−7^	0.9561	10.557·10^−7^	1.9519
Buffer pH 2.0 ^b^					
293.15	1.8538·10^−7^	1.8489·10^−7^	2.6902	1.8386·10^−7^	8.2257
298.15	2.2156·10^−7^	2.2301·10^−7^	−6.5181	2.2353·10^−7^	−8.8859
303.15	2.686·10^−7^	2.6872·10^−7^	−0.4723	2.7002·10^−7^	−5.2979
308.15	3.2558·10^−7^	3.2354·10^−7^	6.2519	3.2418·10^−7^	4.2902
313.15	3.8771·10^−7^	3.8914·10^−7^	−3.691	3.8694·10^−7^	1.979
Buffer pH 7.4 ^c^					
293.15	1.1952·10^−7^	1.1915·10^−7^	3.1335	1.1936·10^−7^	1.3961
298.15	1.4591·10^−7^	1.4708·10^−7^	−8.0535	1.4693·10^−7^	−7.0446
303.15	1.8073·10^−7^	1.7998·10^−7^	4.1779	1.7964·10^−7^	6.0225
308.15	2.1892·10^−7^	2.1840·10^−7^	2.3701	2.1821·10^−7^	3.2370
313.15	2.6240·10^−7^	2.6294·10^−7^	−2.0585	2.6341·10^−7^	−3.8416
Hexane					
293.15	5.5224·10^−5^	5.5187·10^−5^	0.6774	5.5171·10^−5^	0.9647
298.15	7.0509·10^−5^	7.0549·10^−5^	−0.5658	7.0584·10^−5^	−1.0607
303.15	8.9532·10^−5^	8.9506·10^−5^	0.2907	8.9572·10^−5^	−0.4460
308.15	11.287·10^−5^	11.274·10^−5^	1.1049	11.279·10^−5^	0.6517
313.15	14.101·10^−5^	14.103·10^−5^	−0.1695	14.099·10^−5^	0.1353
Ethanol					
293.15	0.9236·10^−2^	0.9240·10^−2^	0.0023	0.9213·10^−2^	2.9608
298.15	1.1339·10^−2^	1.1326·10^−2^	1.2112	1.1348·10^−2^	−0.7125
303.15	1.3831·10^−2^	1.3835·10^−2^	−0.3595	1.3883·10^−2^	−3.8107
308.15	1.6854·10^−2^	1.6844·10^−2^	0.3442	1.6873·10^−2^	−1.3468
313.15	2.0450·10^−2^	2.0441·10^−2^	0.4360	2.0379·10^−2^	3.4562
1-Propanol					
293.15	7.9034·10^−3^	7.8833·10^−3^	2.1086	7.8924·10^−3^	0.9592
298.15	9.5491·10^−3^	9.5597·10^−3^	−6.2862	9.5750·10^−3^	−2.6146
303.15	11.557·10^−3^	11.528·10^−3^	6.2322	11.542·10^−3^	1.5228
308.15	13.829·10^−3^	13.827·10^−3^	−1.9602	13.830·10^−3^	0.0031
313.15	16.474·10^−3^	16.501·10^−3^	−0.0372	16.475·10^−3^	−0.3328
1-Octanol					
293.15	6.2145·10^−3^	6.2160·10^−3^	−0.9679	6.2245·10^−3^	−2.3294
298.15	7.5228·10^−3^	7.4880·10^−3^	4.2499	7.4831·10^−3^	4.9131
303.15	8.9147·10^−3^	8.9532·10^−3^	−4.8528	8.9416·10^−3^	−3.5518
308.15	10.657·10^−3^	10.629·10^−3^	2.9069	10.623·10^−3^	3.4729
313.15	12.526·10^−3^	12.534·10^−3^	−0.3032	12.551·10^−3^	−1.6928

^a^ Composition of aqueous buffer pH 1.2: KCl (3.73 g in 1 L) and 0.1 mol·L^−1^ hydrochloric acid (850 mL in 1 L); ^b^ composition of aqueous buffer pH 2.0: KCl (6.57 g in 1 L) and 0.1 mol·L^−1^ hydrochloric acid (119.0 mL in 1 L); ^c^ composition of aqueous buffer pH 7.4: KH_2_PO_4_ (9.1 g in 1 L) and Na_2_HPO_4_•12H_2_O (23.6 g in 1 L); standard uncertainties: *u*(*T*) = 0.15 K and *u*(*p*) = 3 kPa. Relative standard uncertainties for solubility: *u_r_(x)* = 0.045 for buffer solutions and *u_r_*(*x*) = 0.04 for hexane and 1-octanol.

**Table 5:** Parameters of modified Apelblat and van’t Hoff equations for of IVZ in the selected solvents.

Solvents	*A*	*B*	*C*	*RMSD*	10^3^ *RAD*
Modified Apelblat equation					
Buffer pH 1.2	−34.28	−788.9	4.01	1.43·10^−9^	1.3
Buffer pH 2.0	−133.86	2450.3	19.36	1.31·10^−9^	3.9
Buffer pH 7.4	41.51	−5666.2	−6.71	7.28·10^−10^	3.8
Hexane	−7.99	−3725.7	1.92	5.29·10^−9^	0.6
Ethanol	−72.53	−22.3	11.96	8.08·10^−6^	0.5
1-Propanol	−12.03	2544.3	2.79	4.43·10^−5^	3.3
1-Octanol	39.18	−4720.7	−4.96	2.84·10^−5^	3.6
van’t Hoff equation					
Buffer pH 1.2	−7.36	−2003.4		1.69·10^−9^	1.7
Buffer pH 2.0	−3.86	−3415.4		1.47·10^−9^	5.7
Buffer pH 7.4	−3.55	−3633.4		8.70·10^−10^	4.3
Hexane	4.88	−4306.6		5.62·10^−8^	0.7
Ethanol	7.74	−3644.2		4.27·10^−5^	2.5
1-Propanol	6.68	−3378.1		4.49·10^−5^	4.1
1-Octanol	5.90	−3219.1		2.97·10^−5^	3.7

**Table 6:** Temperature dependences of ideal solubility (ln*x_id_*) and activity coefficients at infinite dilution (ln*γ^∞^*) of IVZ in the studied solvents at *p* = 101.3 kPa.

*T*/K	ln*x*_id_	ln*γ^∞^*						
		Buffer pH 1.2	Buffer pH 2.0	Buffer pH 7.4	Hexane	Ethanol	1-Propanol	1-Octanol
293.15	−2.68	11.51	12.82	13.26	7.12	2.00	2.16	2.40
298.15	−2.53	11.55	12.80	13.21	7.03	1.95	2.12	2.36
303.15	−2.37	11.59	12.76	13.15	6.95	1.91	2.09	2.34
308.15	−2.22	11.64	12.72	13.11	6.86	1.86	2.06	2.32
313.15	−2.08	11.67	12.69	13.08	6.79	1.81	2.03	2.30
^a^ A		14.15 ± 0.05	10.69 ± 0.1	10.34 ± 0.1	1.91 ± 0.04	−0.96 ± 0.02	0.11 ± 0.06	0.91 ± 0.11
^a^ B		−774 ± 15	625 ± 31	854 ± 18	1526 ± 13	867 ± 5	598 ± 16	435 ± 33
^b^ R		0.9994	0.9962	0.9962	0.9999	0.9999	0.9988	0.9913

The standard uncertainties are *u(T)* = 0.15 K, *u*(*p*) = 3 kPa; the relative standard uncertainty is *u_r_*(*γ^∞^*) = 0.04, ^a^ parameters of the correlation equation: ln*γ^∞^* = A + B/(*T*/K); ^b^ R is the pair correlation coefficient.

**Table 7:** Excess thermodynamic solubility functions of IVZ in studied solvents at 298.15 K and *p* = 101.3 kPa.

Solvent	*G^E^*, kJ·mol^−1^	*H^E^*, kJ·mol^−1^	*TS^E^*, kJ·mol^−1^	*S^E^*, J·mol^−1^·K^−1^	^a^ ς_H_^E^, %	^b^ ς_TS_^E^,%
Buffer pH 1.2	28.6 ± 0.5	−6.43 ± 0.1	−35.03	−117.5 ± 4.1	15.3	84.7
Buffer pH 2.0	31.7 ± 0.6	5.20 ± 0.3	−26.50	−88.9 ± 5.1	16.4	83.6
Buffer pH 7.4	32.7 ± 0.6	7.10 ± 0.1	−25.6	−85.9 ±2.9	21.7	78.3
Hexane	17.4 ± 0.3	12.69 ± 0.1	−4.71	−15.8 ± 0.4	72.9	27.1
Ethanol	4.83 ± 0.1	7.21 ± 0.1	2.38	8.0 ± 0.3	75.2	24.8
1-Propanol	5.25 ± 0.2	4.97 ± 0.1	−0.28	−0.9 ± 0.04	94.7	5.3
1-Octanol	5.85 ± 0.2	3.62 ± 0.1	−2.23	−7.5 ± 0.3	61.9	38.1

^a^$\varsigma_{H}=(H^{E}/(H^{E}+TS^{E}))100\%$; ^b^$\varsigma_{TS}=(TS^{E}/(H^{E}+TS^{E}))100\%$; relative standard uncertainties are *u*(*T*) = 0.05 K, *u*(*p*) = 3 kPa; uncertainties for the *G^E^*, *H^E^* and *S^E^* values represent two standard deviations.

**Table 8:** Experimental concentrations, partition coefficients and transfer thermodynamic parameters of IVZ in the 1-octanol/buffer pH 7.4 system at different temperatures and pressure *p* = 0.1 MPa.

*T*/K	*s_O_* · 10^3^	*s_B_* · 10^6^	*P_O/B_*	log*P_O/B_*	*xo* · 10^4^	*x_B_* · 10^7^	logP*_O/B_
293.15	2.15	6.85	313.71	2.50	3.39	1.22	3.44
298.15	2.15	5.98	359.18	2.56	3.40	1.07	3.50
303.15	2.15	5.20	413.63	2.62	3.42	0.93	3.56
308.15	2.15	4.53	474.77	2.68	3.43	0.81	3.62
313.15	2.15	4.02	535.22	2.73	3.45	0.72	3.68
*A* ^a^	16.52 ± 0.09		$\Delta_{tr}G_{}^{{}^{\circ}}$ = −19.99 ± 0.4/kJ·mol^−1^				
*B* ^a^	2520 ± 26		$\Delta_{tr}H_{}^{{}^{\circ}}$ = 20.95 ± 0.2/kJ·mol^−1^				
*R* ^b^	0.9998		$T\Delta_{tr}S_{}^{{}^{\circ}}$ = 40.9 ± 1.5/kJ·mol^−1^				
*σ* ^c^	0.8·10^−2^		$\Delta_{tr}S_{}^{{}^{\circ}}$ = 137.3 ± 5.2/J·mol^−1^K^−1^				

^a^ Parameters of the correlation equation: ln*P_O/B_* = *A* − *B*/*T*; ^b^ *R* is the pair correlation coefficient; ^c^ *σ* is the standard deviation.

**Table 9:** Sample table.

Chemical Name	CAS Register No.	Formula	M/g mol^−1^	Source	Initial Mass Fraction Purity	Final Mass Fraction Purity
Isavuconazole	241479-67-4	C_22_ H_17_ F_2_ N_5_ O S	437.5	Shanghai Send Pharmaceutical Technology	≥0.99 ^a^	No purification
Ethanol	64-17-5	C2H6O	46.1	Sigma-Aldrich	≥0.99 ^b^	No purification
1-Propanol	71-23-8	C3 H8 O	60.1	Sigma-Aldrich	≥0.99 ^b^	No purification
1-Octanol	111-87-5	C_8_H_18_O	130.2	Sigma-Aldrich	≥0.99 ^b^	No purification
Hexane	110-54-3	C_6_H_14_	86.2	Sigma-Aldrich	≥0.97 ^b^	≥0.98 ^c^
Potassium dihydrogen phosphate	7778-77-0	KH_2_PO_4_	136.1	Merck	≥0.99 ^c^	No purification
Disodium hydrogen phosphate dodecahydrate	10039-32-4	Na_2_HPO_4_·12H_2_O	358.1	Merck	≥0.99 ^b^	No purification
Potassium chloride	7447-40-7	KCl	74.5	Sigma-Aldrich	≥0.99 ^b^	No purification
Hydrochloric acid mol/dm^3^ fixanal	7647-01-0	Aldrich	-	None		No purification

^a^ Accordance with Certificate of Analysis (Figure S6); ^b^ as stated by the supplier; ^c^ purified by rectification and analyzed by HPLC.

## Data Availability

Not applicable.

## Supplementary Materials

The following are available online, Figure S1: Correlation sublimation Gibbs energy and HYBOT descriptors for the test set of IVZ: (a)—molecular polarizability; (b)—total acceptor ability. Figure S2: Correlation between Gibbs energy and enthalpy of sublimation for the test set of IVZ. Figure S3: Correlation sublimation Gibbs energy and HYBOT descriptors for the test set of bicalutamide: (a)—molecular polarizability; (b)—total acceptor ability. Figure S4: Correlation between Gibbs energy and enthalpy of sublimation for the test set of bicalutamide. Figure S5: PXRD patterns of raw and equilibrate IVZ from the selected solvents. Figure S6: Analysis certificate of IVZ. Figure S7: Experimental vapor pressures of the benzoic acid. Figure S6: UV–visible absorption spectra of IVZ in organic solvents. Table S1: Cluster of structurally related compounds for IVZ. Table S2: Cluster of structurally related compounds for bicalutamide. Table S3: Group contribution parameters and associated molar volume of IVZ. Table S4: Density of the investigated solvents at different temperatures and pressure *p* = 0.1 MPa.

## Sample Availability

Samples of the compound isavuconazole (Shanghai Send Pharmaceutical Technology, Purity ≥ 0.99) are available from the authors.
