# Supplementary material for: Isavuconazole: Thermodynamic Evaluation of Processes Sublimation, Dissolution and Partition in Pharmaceutically Relevant Media
Source: Molecules. 2021 Aug 6;26(16):4759. doi: 10.3390/molecules26164759 (PMC8398847; doi:10.3390/molecules26164759)
Supplement: Supplementary file 1 [file molecules-26-04759-s001.zip › molecules-1316627-supplementary.pdf]

Supplementary material

for

Isavuconazole: thermodynamic evaluation of processes sublimation, dissolution and partition in pharmaceutically relevant media

Marina V. Ol'khovich, Angelica V. Sharapova, Svetlana V. Blokhina, German L. Perlovich*

Institute of Solution Chemistry, Russian Academy of Sciences,

1 Akademicheskaya Street, 153045, Ivanovo, Russia

Table S1. Cluster of structurally related compounds for drug studied (IVZ)

№	Name	CAS number	$\Delta_{cr}^{g}H_{m}^{o}$ kJ·mol^{-1}	$\Delta_{cr}^{g}G_{m}^{o}$ kJ·mol^{-1}	α A^3	ΣC$_a$	ΣC$_d$	Σ(C$_a$)/α	ΣC$_{ad}$	Σ(C$_d$)/α	Σ(C$_{ad}$)/α	Ref.
1	Flurbiprofen	5104-49-4	110.2	53.3	25.936	5.67	-2.82	0.219	8.49	-0.109	0.327	[1-2]
2	2-fluorobenzoic acid;	445-29-4	94.4	35.5	12.606	4.14	-3.15	0.328	7.29	-0.25	0.579	[3]
3	3-fluorobenzoic acid	455-38-9	93.6	34.4	12.606	4.14	-2.89	0.328	7.03	-0.229	0.557	[3]
4	4-fluorobenzoic acid	456-22-4	93.1	36.8	12.606	4.21	-2.89	0.334	7.1	0.234	0.563	[4-5]
5	2,2'-difluorobiphenyl	388-82-9	95.0	32.3	19.912	1.04	0	0.052	1.04	0	0.052	[6]
6	4,4'-difluorobiphenyl	398-23-2	91.2	30.2	19.912	1.13	0	0.057	1.13	0	0.057	[6]
7	1-methyl-1-phenylethanol	617-94-7	82.3	22.0	16.576	1.83	-1.87	0.111	3.7	-0.11	0.223	[7]
8	1,1-diphenylethanol	599-67-7	105.0	39.1	24.401	2.13	-1.87	0.087	4	-0.07	0.164	[8]
9	4-Fluorophenol	371-41-5	74.1	19.9	10.617	1.11	-2.7	0.104	3.8	-0.254	0.358	[9]
10	2-Naphthalenecarbonitrile	613-46-7	92.1	33.8	19.564	2.54	0	0.13	2.54	0	0.13	[10]
11	Bicalutamide	90357-06-5	124.7	63.7	36.871	8.1	-4.4	0.22	12.49	-0.119	0.339	[11]
12	3-Fluorobenzonitrile	403-54-3	65.4	15.8	12.203	1.72	0	0.141	1.72	0	0.141	[12]
13	4-Fluorobenzonitrile	1194-02-1	67.7	17.6	12.203	1.87	0	0.153	1.87	0	0.15	[12]
14	4-Iodobenzonitrile	3058-39-7	88.0	32.8	17.322	1.9	0	0.11	1.9	0	0.11	[13]
15	4-Bromobenzonitrile	623-00-7	80.8	28.5	14.92	1.83	0	0.123	1.83	0	0.123	[14]
16	4-chlorobenzonitrile	623-03-0	76.7	23.5	14.222	1.86	0	0.131	1.86	0	0.131	[15]
17	*p*-fluoroacetophenone	403-42-9	71.6	17.6	14.167	1.95	0	0.138	1.95	0	0.138	[16]
18	Isavuconazole	241479-67-4	138.1	68.9	44.097	8.14	-1.87	0.185	10	-0.042	0.227	Our study

Thermodynamic functions have been obtained at 298.15 K

References

1. Perlovich, G.L.; Kurkov, S.V.; Bauer-Brandl, A. Thermodynamics of Solutions II: Flurbiprofen and Diflunisal as Models for Studying Solvation of Drug Substances. *Eur. J. Pharm. Sci.* **2003**, 19, 423-432. https://doi.org/10.1016/S0928-0987(03)00145-3.

2. Kurkov, S.V., Perlovich, G.L. Thermodynamic studies of Fenbufen, Diflunisal, and Flurbiprofen:Sublimation, solution and solvation of biphenyl substituted drugs. *Int. J. Pharmaceut*. **2008**, 357, 100–107. https://doi.org/10.1016/j.ijpharm.2008.01.059.

3. Monte, M.J S., Hillesheim, D.M., Thermodynamic study on the sublimation of the three iodobenzoic acids and of 2-fluoro- and 3-fluorobenzoic acids. *J. Chem. Thermodyn*. **2000**, 32, 1727–1735. https://doi.org/10.1006/jcht.2000.0705.

4. Cox, J.D., Gundry, H.A., Harrop, D., Head, A.J. Thermodynamic properties of fluorine compounds. 9. Enthalpies of formation of some compounds containing the pentafluorophenyl group. *J. Chem. Thermodyn*. **1969**, 1, 77–87. https://doi.org/10.1016/0021-9614(69)90038-X.

5. J. D. Cox and G. Pilcher, Thermochemistry of Organic and Organometallic Compounds (Academic, New York, 1970).

6. Smith N.K., Gorin G., Good W.D., McCullough J.P., The Heats of Combustion, Sublimation, and Formation of Four Dihalobiphenyls. *J. Phys. Chem. A*. **1964**, 68, 940–946. https://doi.org/10.1021/j100786a043

7. Verevkin, S.P. Thermochemical Properties of Triphenylalkanes and Tetraphenylmethane. Strain in Phenyl Substituted Alkanes. *J. Chem. Eng. Data.* **1999**, 44, 1245-1251. https://doi.org/10.1021/je9802726.

8. Verevkin, S.P., Thermochemistry of Alcohols: Experimental Standard Molar Enthalpies of Formation and Strain of Some Alkyl and Phenyl Congested Alcohols *Struct. Chem*. **1998**, 9, 375-382. https://doi.org/10.1023/A:1022471228499

9. Almeida, A.R.R.P., Monte, M.J.S. Crystalline and liquid vapour pressures of the four p-monohalophenols: A thermodynamic study of their phase transitions. *J. Chem. Thermodyn.* **2013**, 65, 150–158. https://doi.org/10.1016/j.jct.2013.05.047.

10. Ribeiro da Silva, M.A.V., Lobo Ferreira, A.I.M.C., Barros, A.L.M., Bessa, A.R.C., Brito, B.C.S.A., Vieira, J.A.S., Martins, S.A.P. Standard molar enthalpies of formation of 1- and 2-cyanonaphthalene. *J. Chem. Thermodyn.* **2011**, 43, 1306–1314. https://doi.org/10.1006/jcht.2001.0905.

11. Perlovich, G.L., Blokhina, S.V., Manin, N.G., Volkova, T.V., Tkachev, V.V. Polymorphism and solvatomorphism of bicalutamide. *J. Therm. Anal. Calorim*. **2013**, 111, 655–662. https://doi.org/10.1007/s10973-012-2540-y.

12. Ribeiro da Silva, M.A.V., Monte, M.J.S., Rocha, I.M., Cimas, A. Energetic Study Applied to the Knowledge of the Structural and Electronic Properties of Monofluorobenzonitriles. *J. Org. Chem.* **2012**, 77, 4312-4322. https://doi.org/10.1021/jo3002968.

13. Rocha, I.M., Fialho, R.L., Marques, J.J., Dariva, C., Pesso, F.L.P. Effect of water content on the equilibrium pressure of (carbon dioxide + decane and + decalin) from T = (313.15 to 333.15) K. *J. Chem. Thermodyn.* **2013**, 65, 11-17. https://doi.org/10.1016/j.jct.2013.05.015.

14. Rocha, I.M., Galva, T.L.P., Ribeiro da Silva, M.D.M.C., Ribeiro da Silva, M.A.V. Energetic study of bromobenzonitrile isomers: insights on the intermolecular interactions, aromaticity and electronegativity. *Struct. Chem.* **2013**, 24, 1935–1944. https://doi.org/10.1007/s11224-013-0278-1.

15. Rocha, I.M. Thermodynamic Study of Chlorobenzonitrile Isomers: A Survey on the Polymorphism, Pseudosymmetry, and the Chloro···Cyano Interaction. *J. Phys. Chem. A.* **2014**, 118, 1502-1510. https://doi.org/10.1021/jp410187q.

16. Almeida, A., Monte, M. The influence of the halogen atoms and acetyl group on vapour pressures and related properties of the p-haloacetophenones. *J. Chem. Thermodynam.* **2016,** 92, 118–125. https://doi.org/10.1016/j.jct.2015.09.004.

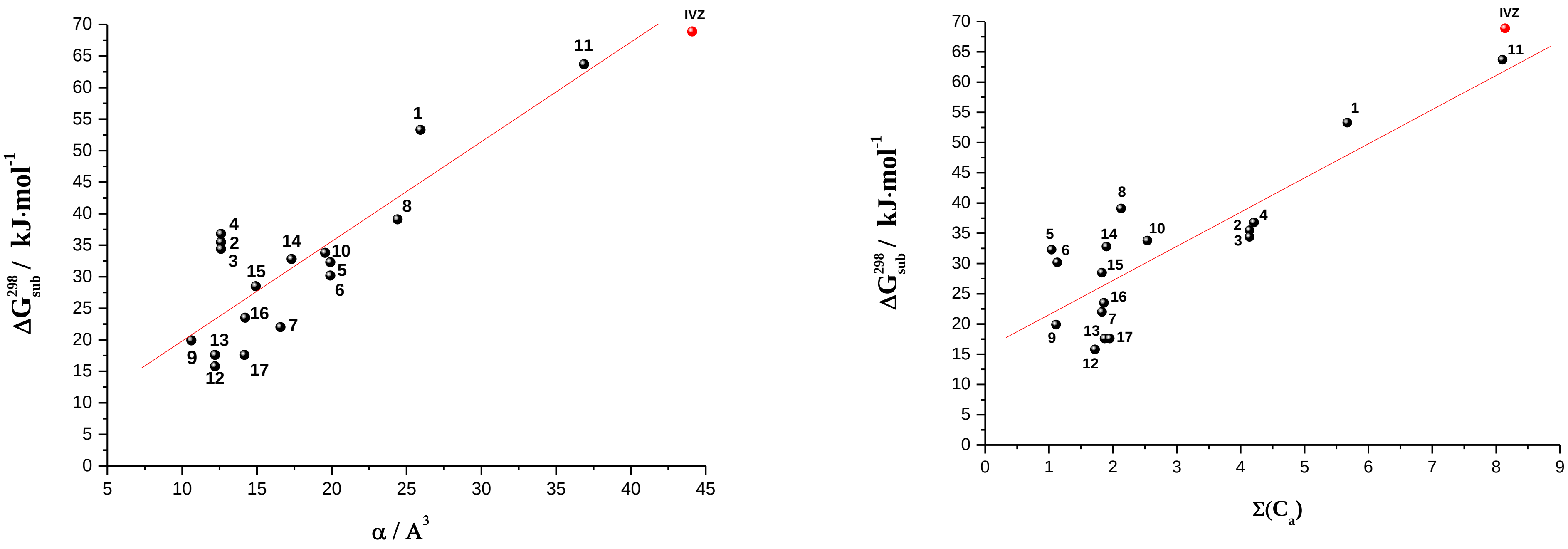

Figure S1. Correlation sublimation Gibbs energy and HYBOT descriptors for the test set of IVZ: a) - molecular polarizability; b) - total acceptor ability.

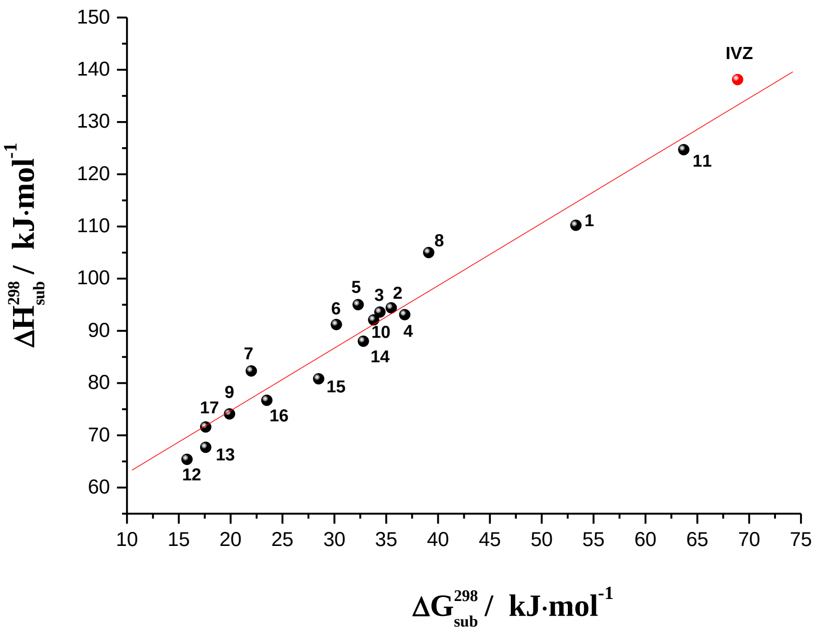

Figure S2. Correlation between Gibbs energy and enthalpy of sublimation for the test set of IVZ.

The analogous analysis for estimation of the sublimation thermodynamic functions of Isavuconazole (IVZ) with applying database clusterization is presented for Bicalutamide (as an ancestor of the cluster). It was selected 45 structural relative compounds to Bicalutamide with 0.75 < Tc < 1 (Isavuconazole was excluded from this set). The results of the clusterization are shown in Table S2. Correlations between sublimation Gibbs energy and HYBOT descriptors for the test set of Bicalutamide: a) - molecular polarizability; b) - total acceptor ability are presented in Figure S3.

Within the formed cluster for Bicalutamide, we tried to find a correlation between the Gibbs energies of sublimation $\Delta_{cr}^{g}G_{m}^{0}(298.15K)$ and the physicochemical descriptors of HYBOT. Using the entire set of descriptors, the best correlations were observed for molecular polarizability (α) (Figure S3a) and total acceptor ability of a molecule to form hydrogen bonds (Σ(Ca)) (Figure S3b). As a result, a two-parameter correlation equation was obtained to estimate $\Delta_{cr}^{g}G_{m}^{0}(298.15K)$:

$$\Delta_{cr}^{g}G_{m}^{o}(298.15K) = (17.89 \pm 3.74) + (0.380 \pm 0.209)\cdot\alpha + (5.384 \pm 0.674)\cdot\Sigma(C_{a})$$

R = 0.8475; SD = 7.26 kJ·mol^{-1}; n = 44; F = 53.5

In order to obtain a complete thermodynamic picture of IVZ sublimation process, it was necessary to estimate the value of enthalpy. For this purpose, we used the correlation dependence between the Gibbs energies and the enthalpies of sublimation (the so-called "compensation effect") within the selected cluster (Figure S4). As a result, the following equation was obtained:

$$\Delta_{cr}^{g}H_{m}^{0}(298.15K) = (45.5 \pm 3.5) + (1.329 \pm 0.073)\cdot\Delta_{cr}^{g}G_{m}^{0}(298.15K)$$

R = 0.9414; SD = 6.43 kJ·mol-1; n = 45;

The sublimation thermodynamic functions of IVZ calculated from these equations are in good agreement with the experimental values: $\Delta_{cr}^{g}G_{m}^{0}(298.15K)_{\text{cal}}$ = 78.5 kJ·mol^{-1} and $\Delta_{cr}^{g}G_{m}^{0}(298.15K)_{\text{exp}}$= 68.9 ± 0.2 kJ·mol^{-1}; $\Delta_{cr}^{g}H_{m}^{0}(298.15K)_{\text{cal}}$ = 149.8 kJ·mol^{-1} and $\Delta_{cr}^{g}H_{m}^{0}(298.15K)_{\text{exp}}$ = 138.1 ± 0.5 kJ·mol^{-1}.

Table S2. Cluster of structurally related compounds for compound Bicalutamide

№	Name	CAS number	$\Delta_{cr}^{g}H_{m}^{o}$ kJ·mol^{-1}	$\Delta_{cr}^{g}G_{m}^{o}$ kJ·mol^{-1}	α A^{3}	ΣC_a	ΣC_d	ΣC_{ad}	$\Sigma(C_a)/\alpha$	$\Sigma(C_d)/\alpha$	$\Sigma(C_{ad})/\alpha$
1	Acetamide	103-84-4	99.8	40.5	15.735	3.25	-1.97	5.22	0.206	-0.125	0.331
2	Paracetamol	103-90-2	117.9	60	16.009	4.15	-4.53	8.68	0.259	-0.283	0.542
3	Phenacetine	62-44-2	121.8	52.3	19.679	3.75	-1.9	5.65	0.191	-0.097	0.287
4	Mefenamic Acid	61-68-7	136.3	59.2	27.504	6.02	-4.49	10.52	0.219	-0.163	0.382
5	Flufenamic acid	530-78-9	121.2	54.3	25.396	5.47	-4.4	9.87	0.215	-0.173	0.389
6	Niflumic acid	4394-00-7	130.2	61.3	24.687	6.52	-5.16	11.68	0.264	-0.209	0.473
7	N-(2-methylphenyl)acetamide	120-66-1	97.1	40.9	17.57	3.59	-2.53	6.12	0.205	-0.144	0.349
8	N-(4-methylphenyl)acetamide	103-89-9	99	44.5	17.57	3.14	-1.9	5.04	0.179	-0.108	0.287
9	p-acetaminobenzaldehyde	122-85-0	99.1	44.1	17.724	4.23	-1.9	6.13	0.238	-0.107	0.346
10	benzanilide	93-98-1	99.2	51.3	23.56	3.13	-2.53	5.66	0.133	-0.107	0.24
11	N-acetyl-1-naphthylamine	575-36-0	94	47.9	23.005	3.79	-2.53	6.32	0.165	-0.11	0.275
12	2-fluorobenzoic acid	445-29-4	94.4	35.5	12.606	4.14	-3.15	7.29	0.328	-0.25	0.579
13	3-fluorobenzoic acid	455-38-9	93.6	34.4	12.606	4.14	-2.89	7.03	0.328	-0.229	0.557
14	4-fluorobenzoic acid	456-22-4	93.1	36.8	12.606	4.21	-2.89	7.1	0.334	-0.229	0.563
15	2,2'-difluorobiphenyl	388-82-9	95	32.3	19.912	1.04	0	1.04	0.052	0	0.052
16	4,4'-difluorobiphenyl	398-23-2	91.2	30.2	19.912	1.13	0	1.13	0.057	0	0.057
17	a,a-dimethylbenzyl alcohol	617-94-7	82.3	22	16.576	1.83	-1.87	3.7	0.111	-0.113	0.223
18	acetylglycine anilide	34329-64-1	125.4	59.9	22.877	6.56	-4.41	10.97	0.287	-0.193	0.48
19	N-acetyl-1-naphthylamine	575-36-0	94.1	47.9	23.005	3.79	-2.53	6.32	0.165	-0.11	0.275
20	N-phenyl benzylamine	103-32-2	104.4	36.9	23.406	2.42	-1.46	3.88	0.103	-0.062	0.166
21	1,1-diphenylethanol	599-67-7	105	39.1	24.401	2.13	-1.87	4	0.087	-0.077	0.164
22	2'-Hydroxyacetanilide	614-80-2	121.8	52.8	16.009	3.63	-5.53	9.16	0.226	-0.345	0.572
23	3-Acetamidophenol	621-42-1	111	56.4	16.009	4.15	-4.9	9.05	0.259	-0.306	0.565
24	N-phenyl-Anthranilic acid	91-40-7	126	58.9	23.834	5.59	-4.4	9.99	0.235	-0.185	0.419
25	2-(acetylamino)-Benzoic acid	89-52-1	115.5	54.4	17.998	6.82	-5.68	12.51	0.379	-0.316	0.695
26	4-Cyanobenzoic acid	619-65-8	112.8	50.7	14.557	5.14	-2.89	8.03	0.353	-0.199	0.551

27	1-(4-fluorophenyl)pyrrole	81329-31-9	78.9	25.4	18.002	1.46	0	1.46	0.081	0	0.081
28	3-Fluoronitrobenzene	402-67-5	72.3	17.1	12.184	1.32	0	1.32	0.108	0	0.108
29	4-Fluoronitrobenzene	350-46-9	70.9	18.9	12.184	1.44	0	1.44	0.118	0	0.118
30	2-Fluorobenzamide	445-28-3	95.4	35.8	13.809	2.95	-4.16	7.11	0.213	-0.301	0.515
31	3-Fluorobenzamide	455-37-8	104.1	42.5	13.809	2.82	-4.16	6.98	0.204	-0.301	0.505
32	4-Fluorobenzamide	824-75-9	102.8	43.2	13.809	2.91	-4.16	7.07	0.21	-0.301	0.512
33	4-Fluorophenol	371-41-5	74.1	19.9	10.617	1.11	-2.7	3.8	0.104	-0.254	0.358
34	4-(methylamino)benzoic acid	10541-83-0	123.9	55.3	16.009	5.5	-4.37	9.86	0.343	-0.273	0.616
**35**	**Bicalutamide**	**90357-06-5**	**124.7**	**63.7**	**36.871**	**8.1**	**-4.4**	**12.49**	**0.22**	**-0.119**	**0.339**
36	2-phenylcarbamoyl-3-methylquinoxaline-N-oxide		145.1	69.2	32.505	9.01	-2.53	11.54	0.277	-0.078	0.355
37	o-Aminobenzamide	88-68-6	106.8	46.1	15.377	4.23	-5.16	9.39	0.275	-0.336	0.611
38	m-Aminobenzamide	3544-24-9	125.3	57.5	15.377	4.49	-6.29	10.78	0.292	-0.409	0.701
39	m-Aminobenzamide	3544-24-9	128.6	57.8	15.377	4.49	-6.29	10.78	0.292	-0.409	0.701
40	p-Aminobenzamide	2835-68-9	131.2	61.7	15.377	4.46	-6.94	11.4	0.29	-0.451	0.741
41	1-[5-(4-Fluoro-phenylamino)-[1,2,4]thiadiazol-3-yl]-propan-2-ol		124.8	59.1	25.36	5.18	-3.13	8.31	0.204	-0.123	0.328
42	3-methyl-N-(2-methylphenyl)-2-quinoxalinecarboxamide-1,4-dioxide	111888-46-1	154.8	71.9	34.34	9.16	-2.53	11.69	0.267	-0.074	0.341
43	Diflunisal (Form A)	27494-42-4	120.1	57.6	22.449	4.84	-4.85	9.69	0.216	-0.216	0.432
44	4'-Chloroacetanilide	539–03-7	113.8	50.1	17.663	3.16	-2.02	5.18	0.179	-0.114	0.293
45	4'-Bromoacetanilide	103–88-8	113.4	51.2	18.361	3.1	-2.13	5.23	0.169	-0.116	0.285
46	N-(3,5-Dimethyl-adamantan-1-yl)-4-trifluoromethyl-benzenesulfonamide		107.7	49.6	37.009	3.07	-1.74	4.8	0.083	-0.047	0.13
**47**	**Isavuconazole (IVZ)**	**241479-67-4**	**138.1**	**68.9**	**44.097**	**8.14**	**-1.87**	**10**	**0.185**	**-0.042**	**0.227**

Thermodynamic functions have been obtained at 298.15 K

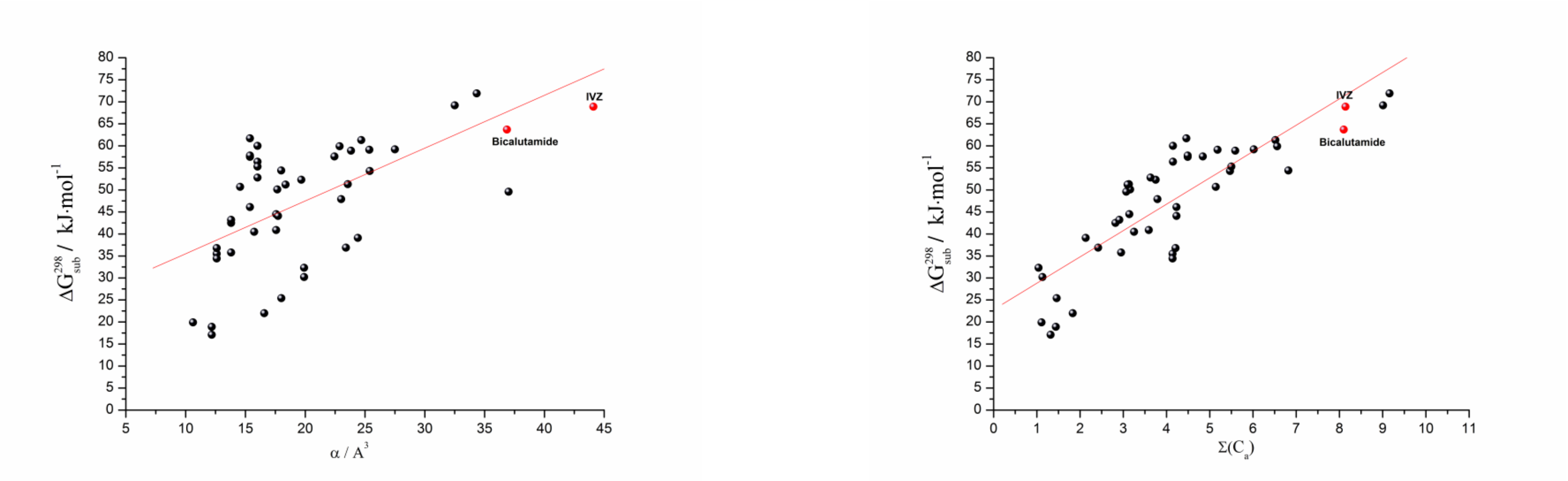

Figure S3. Correlation sublimation Gibbs energy and HYBOT descriptors for the test set of Bicalutamide: a) - molecular polarizability; b) - total acceptor ability.

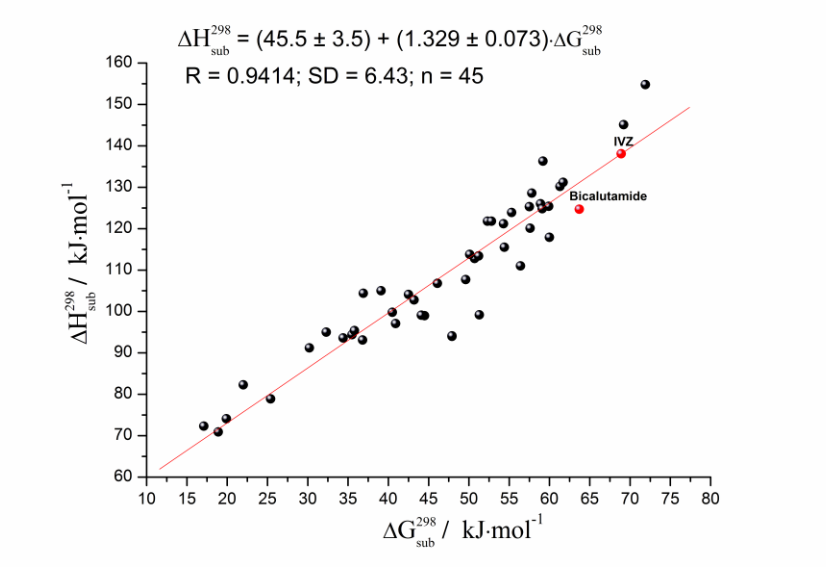

Figure S4. Correlation between Gibbs energy and enthalpy of sublimation for the test set of Bicalutamide.

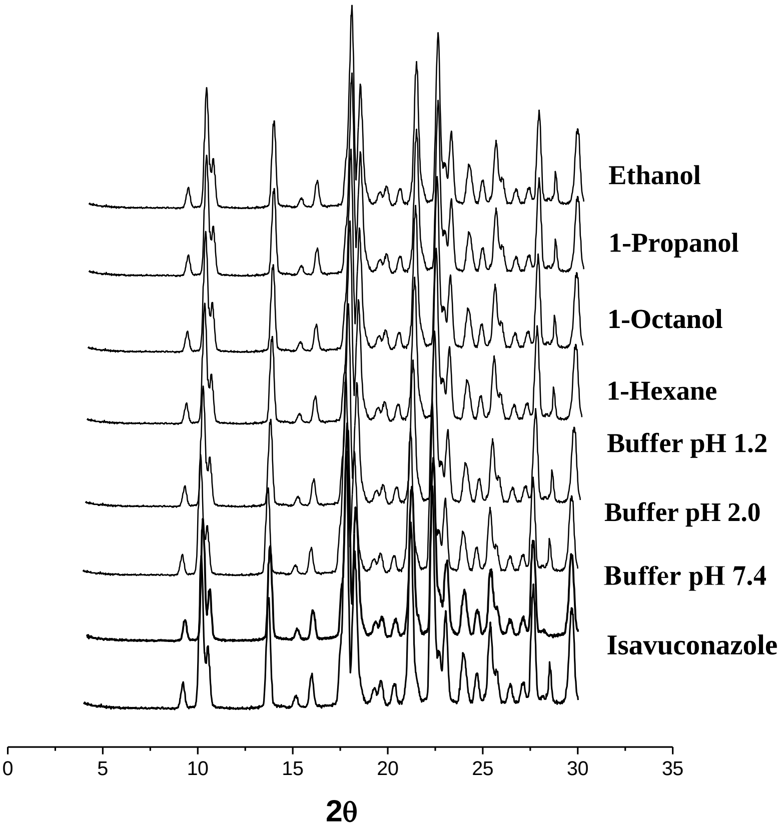

Figure S5. PXRD patterns of raw and equilibrate IVZ from the selected solvents

Table S3. Group contribution parameters and associated molar volume of IZV

Individual functional group	Frequency	F_{di} ,$(J/cm^3)^{0.5}\cdot mol^{-1}$	F_{pi} , $(J/cm^3)^{0.5}\cdot mol^{-1}$	E_{hi}, J/mol	V_i, cm^3/mol
-F	2	102.0	493.9	6544.3	18.0
$-CH_2-$	1	234.6	0	0	16.1
-OH	1	76.5	1225.0	6060.0	10.0
-CH-	1	132.6	0	0	-1
>C<	1	-214.2	0	0	-19.2
=C<	5	-56.7	20.0	0	-5.5
$-CH_3$	1	336.6	0	0	33.5
>N-	1	30	150.0	750.0	-9
=N-	3	380	100	250	5.0
=CH-	10	255.0	38.0	0	13.5
- S-	1	815.9	196.0	297.5	12
phenyl	2	1515.0	50.0	20.9	71.4
Ring closure 5 or more atoms 3	3	142.8	0	0	16
Total		8480.9	3932.7	20987.9	389.7

上海升德医药科技有限公司

SHANGHAI SEND PHARMACEUTICAL TECHNOLOGY CO.,LTD

Building 1,No.800,qingdai road,Pudong,Shanghai 201318,China

Tel:+86-21-50266790 Fax:+86-21-50266790

CERTIFICATE OF ANALYSIS

**Product Name:**<br>**产品名称**	Isavuconazole<br>艾沙康唑	**Batch No.:**<br>**生产批号**	SEND2019813-67-4
**Quantity:**<br>**数量**	500 g	**CAS NO.:**<br>**CAS 号**	241479-67-4
**Manufacture. Date:**<br>**生产日期**	2019.08.13	**Expiry Date:**<br>**到期日期**	2022.08
**Storage:**<br>**贮存**	Preserve In Well-Closed, Light-Resistant and Tight Containers.<br>Store In Cool & Dry Place.<br>保存在密闭、耐光、密封的容器中。存放于阴凉干燥处。		

**ITEMS**<br>**检测项目**	**SPECIFICATION**<br>**产品规格**	**RESULTS**<br>**检测结果**
**Appearance**<br>**外观**	White to yellow solid	Conforms（符合）
**Loss on d Drying**<br>**干燥失重**	≤1.0%	0.1%
**Related substance**<br>**相关物质**	Single Impurity ≤0.5%<br>Total Impurity≤1.0 %	0.06%<br>0.20%
**Assay (HPLC)**<br>**含量**	≥99%	Conforms
**Conclusion:**<br>**结论**	The results conforms with enterprise standard.<br>结果符合企业标准	

QC: [illegible] QA: [illegible]

Figure S6. Analysis certificate of IVZ.

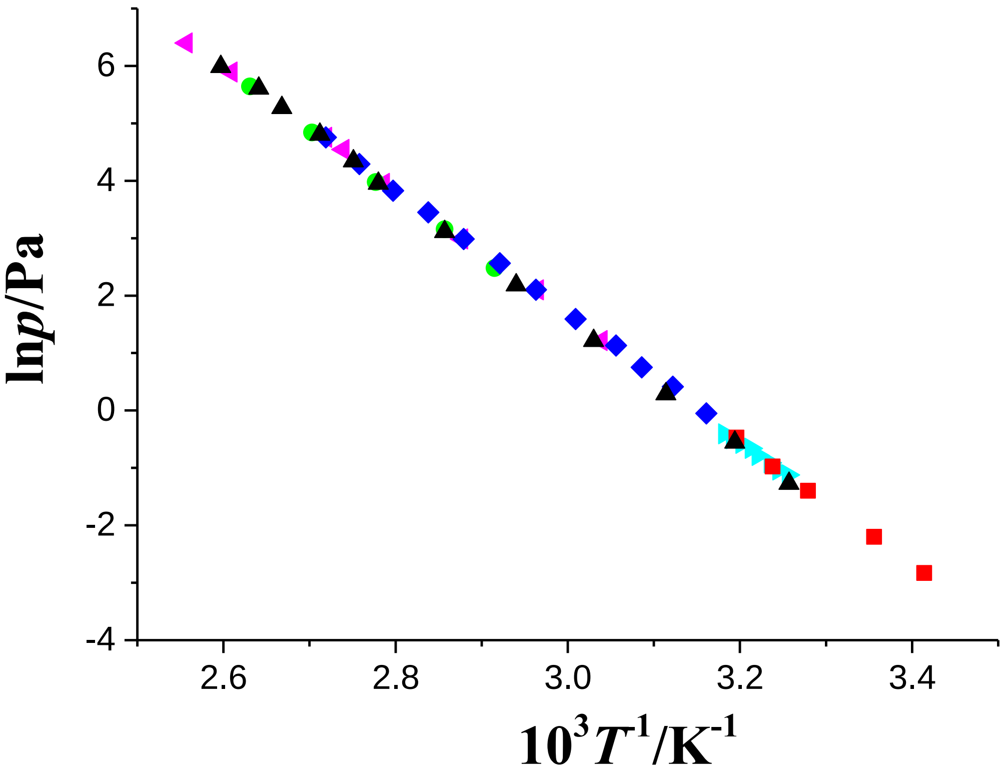

Figure S7. Experimental vapour pressures of the benzoic acid: ▲ − our data; ► − from [Ribeiro da Silva, M.A.V. and etc. Thermochim. Acta. **1990,** *171*, 169–183], ◄ − from [de Kruif, C.G. and etc. J. Chem. Thermodyn. **1982**, *14*, 201–206], ■ − from [Colomina, M. and etc. J. Chem. Thermodyn. **1982**, *14*, 779−784.]; ♦ − from [Verevkin, S.P. and etc. Thermochim. Acta. **2015**, *622*, 18–30.]; ● − from [Davies, M. and etc. Trans. Faraday Soc. **1954,** *50*, 1042–47].

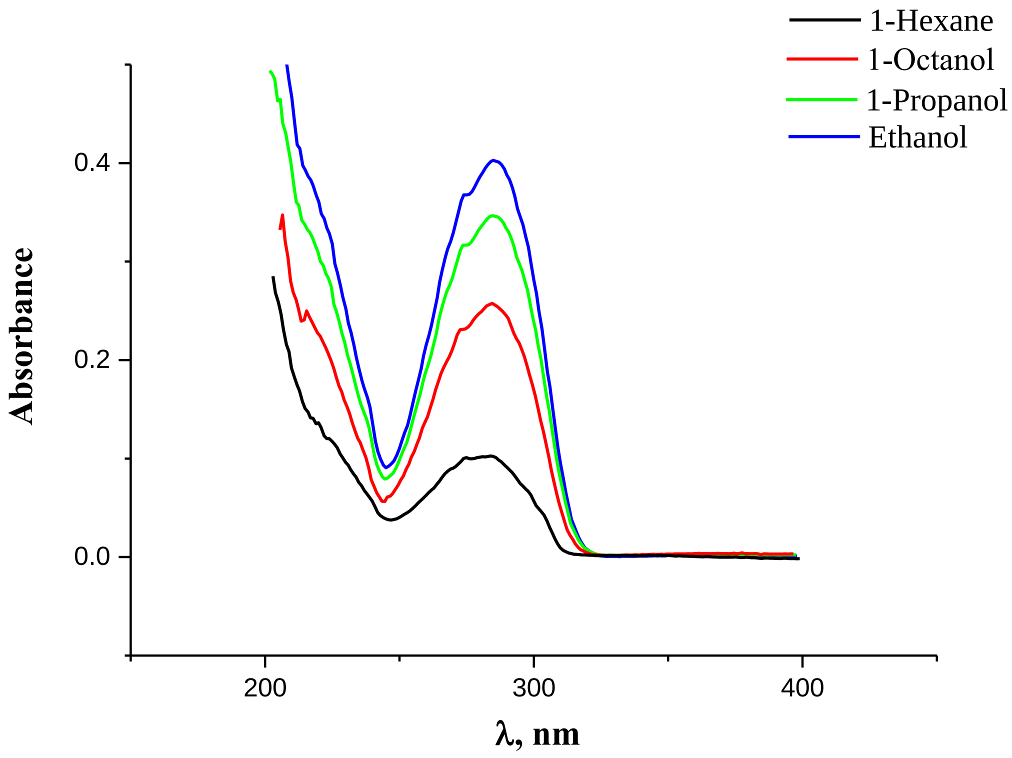

Figure S8. UV–visible absorption spectra of IVZ in organic solvents.

Table S4. Density of the investigated solvents at different temperatures and pressure p = 0.1 MPa

Solvent	ρ/g·cm^{-3}				
	293.15 K	298.15 K	303.15 K	308.15 K	313.15 K
Buffer pH 1.2[a]	1.0022	1.0010	0.9994	0.9979	0.9960
Buffer pH 2.0[b]	1.0035	1.0023	1.0008	0.9992	0.9973
Buffer pH 7.4[c]	1.0060	1.0048	1.0033	1.0016	0.9998
Hexane	0.6587	0.6543	0.6495	0.6453	0.6432
Ethanol	0.7895	0.7852	0.7809	0.7766	0.7722
Propanol	0.8037	0.7995	0.7955	0.7915	0.7874
1-Octanol	0.8251	0.8217	0.8183	0.8148	0.8114

[a]Composition of aqueous buffer pH 1.2: KCl (3.73 g in 1 L) and 0.1 mol·L^{-1} hydrochloric acid (850 mL in 1 L);
[b]Composition of aqueous buffer pH 2.0: KCl (6.57 g in 1 l) and 0.1 mol/dm^3 hydrochloric acid (119.0 ml in 1 l);
[c]Composition of aqueous buffer pH 7.4: KH_2PO4 (9.1 g in 1 l) and $Na_2HPO_4 \cdot 12H_2O$ (23.6 g in 1 l);
Standard uncertainities: $u(m)$=0.01 mg, $u(T)$ = 0.15 K, $u(p)$ = 3 kPa and $u(\rho)$= 0.002 g·cm^{-3}.
